# Arginine methyltransferases PRMT2 and PRMT3 are essential for biosynthesis of plant-polysaccharide-degrading enzymes in *Penicillium oxalicum*

**DOI:** 10.1371/journal.pgen.1010867

**Published:** 2023-07-31

**Authors:** Shuai Zhao, Li-Xiang Mo, Wen-Tong Li, Lian-Li Jiang, Yi-Yuan Meng, Jian-Feng Ou, Lu-Sheng Liao, Yu-Si Yan, Xue-Mei Luo, Jia-Xun Feng

**Affiliations:** State Key Laboratory for Conservation and Utilization of Subtropical Agro-bioresources, Guangxi Research Center for Microbial and Enzyme Engineering Technology, College of Life Science and Technology, Guangxi University, Nanning, People’s Republic of China; Oregon State University, UNITED STATES

## Abstract

Many filamentous fungi produce plant-polysaccharide-degrading enzymes (PPDE); however, the regulatory mechanism of this process is poorly understood. A Gal4-like transcription factor, CxrA, is essential for mycelial growth and PPDE production in *Penicillium oxalicum*. Its N-terminal region, CxrA_Δ207–733_ is required for the regulatory functions of whole CxrA, and contains a DNA-binding domain (CxrA_Δ1–16&Δ59–733_) and a methylated arginine (R) 94. Methylation of R94 is mediated by an arginine N-methyltransferase, PRMT2 and appears to induce dimerization of CxrA_Δ1–60_. Overexpression of *prmt2* in *P*. *oxalicum* increases PPDE production by 41.4–95.1% during growth on Avicel, compared with the background strain Δ*ku70*;*hph*^R+^. Another arginine N-methyltransferase, PRMT3, appears to assist entry of CxrA into the nucleus, and interacts with CxrA_Δ1–60_ *in vitro* under Avicel induction. Deletion of *prmt3* resulted in 67.0–149.7% enhanced PPDE production by *P*. *oxalicum*. These findings provide novel insights into the regulatory mechanism of fungal PPDE production.

## Author summary

Fungal transcription factors (TFs) have important and complex functions in various cellular processes. However, only several TFs have been characterized, hindering a greater understanding of their regulatory functions and especially their detailed mechanisms of action. Plant-polysaccharide-degrading enzymes (PPDEs) are essential for vegetative growth of fungi, because they can degrade complex polysaccharides into monosaccharides, such as glucose, for use as a carbon source. PPDE biosynthesis in fungi is strictly regulated by a complex regulatory network mediated by TFs, but the mechanisms of TF action are poorly understood. This study found that regulation of PPDE biosynthesis by the TF, CxrA in *Penicillium oxalicum* appeared to require a methylated N-terminus, mediated by arginine N-methyltransferase PRMT2 and assistance to enter the nucleus by arginine N-methyltransferase PRMT3, which contributes to a greater understanding of regulation of fungal gene expression and provides potential new targets for genetic engineering.

## Introduction

In nature, plant-polysaccharide-degrading enzymes (PPDE) produced by heterotrophic fungi can efficiently digest plant polysaccharides, including cellulose, xylan and starch, into monosaccharides, which provides a carbon source to support fungal growth and development [1]. However, regulation of PPDE production by fungi is very complex, remaining incompletely understood thus far. Transcription factors (TFs) are centrally involved in this regulation and function at the gene transcription level.

The filamentous fungus *Penicillium oxalicum* is an excellent producer of PPDE that are used as biocatalysts for biorefining of renewable lignocellulosic biomass, to produce biologically-based chemicals, including biofuels. Several TFs have been identified as being involved in control of PPDE biosynthesis in *P*. *oxalicum* [1], including CxrA [2]. CxrA dynamically regulates the expression of genes encoding major PPDEs, such as *cbh1*, *eg1*, *bgl1* and *xyn11A*, as well as regulatory genes, such as *clrB*, *cxrC* and *cbh* [3–5]. The minimal DNA binding domain, CxrA_Δ1–16&59–733_ was found to bind the core DNA sequences 5’-ATCAGATCCTCAAAGA-3’ and 5’-GCTGAGTCCTT-3’ in the promoters of *cbh1* and *clrB*, respectively [4]. However, the mechanism of CxrA function remains to be fully elucidated, specifically its interaction partners and post-translational modification.

Protein methylation is carried out by methyltransferases, which commonly methylate nitrogen atoms in the ε-amino group of lysine and the guanidino group of arginine, respectively, using S-adenosyl-L-methionine as cofactor [6]. Arginine methylation is implicated in fundamental cellular processes, including DNA transcription, splicing and repair, as well as cellular metabolism [7]. Arginine methylation is catalyzed by protein arginine methyltransferase (PRMT), which is generally classified into four types. Type I and II are responsible for biosynthesis of asymmetric and symmetric *ω*-*N*^G^,*N*^G^-dimethylarginine, respectively. Type III is responsible for biosynthesis of *ω*-*N*^G^ monomethyl arginine only, and type IV is for *δ*-*N*^G^ monomethyl arginine; type IV is also specific to fungi [8]. In *Aspergillus nidulans*, *Aspergillus flavus* and *Penicillium expansum*, four PRMTs have been identified, i.e., PRMT1 (type I), PRMT3 (type I), PRMT5 (type II) and RMT2 (type IV). Of these, PRMT1, PRMT3 and PRMT5 are involved in fungal growth, development, stress responses and secondary metabolism [9–11]. However, the effects of these PRMTs on cellulase and xylanase biosynthesis in filamentous fungi have not been reported.

In this study, the molecular mechanism of CxrA regulation was comprehensively elucidated. Notably, methylation of CxrA by PRMT was essential for the proper function in positively regulating the biosynthesis of cellulase and xylanase in *P*. *oxalicum*.

## Results

### N-terminal residues 1–206 are required for the proper function of full-length CxrA

**Fig 1:**
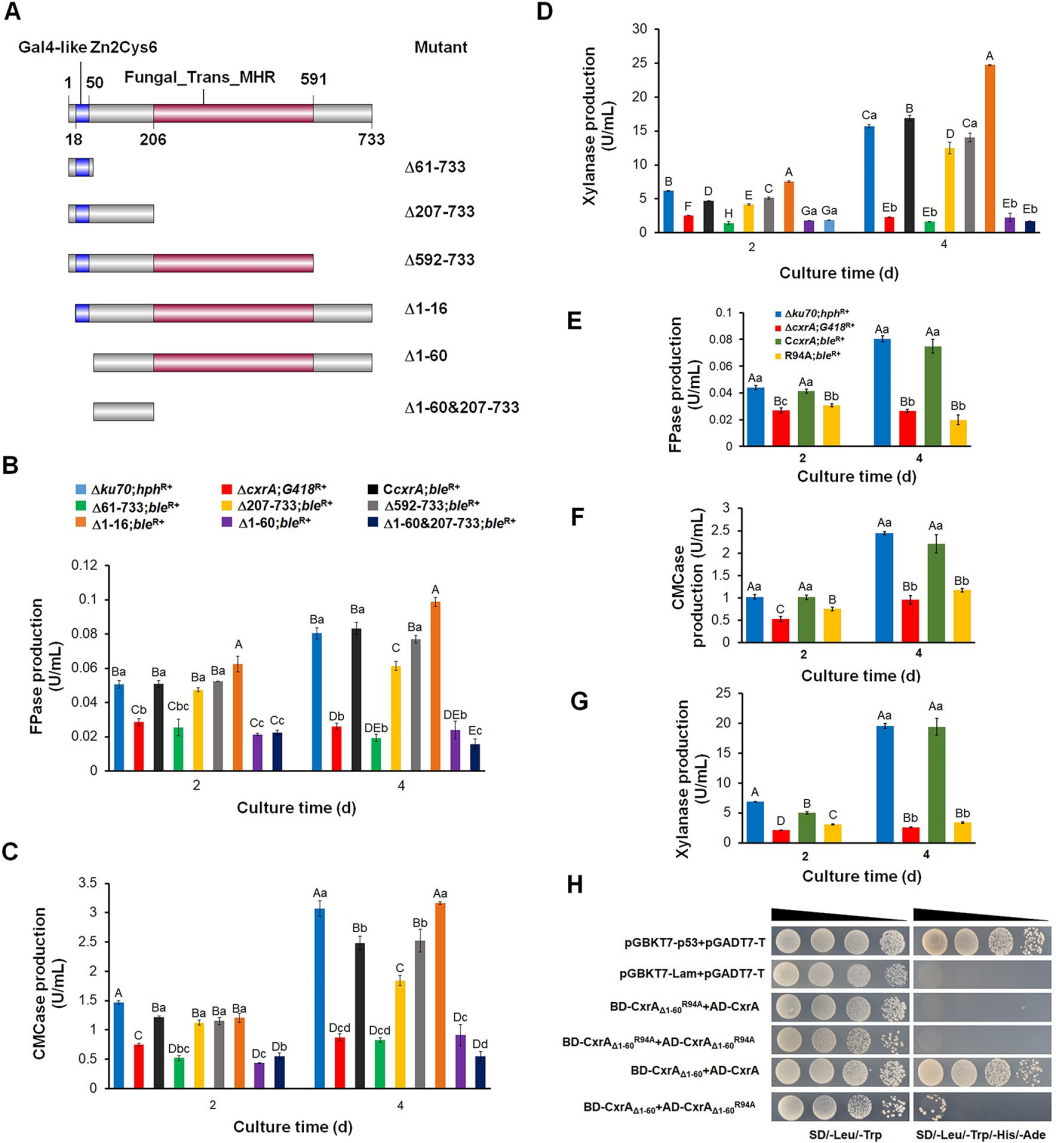
Influences of different regions (A–D) and arginine (R) 94 (E–H) of CxrA on cellulase and xylanase production in *P*. *oxalicum*. (**A**) Construction scheme of *P*. *oxalicum* mutants expressing sequences encoding different regions of CxrA. (**B**) Production of filter paper cellulase (FPase), carboxymethyl cellulase (CMCase) (**C**) and xylanase (**D**) by the constructed *P*. *oxalicum* mutants shown in panel A. (**E**) Effects of R94 on the production of FPase, CMCase (**F**) and xylanase (**G**). Mutant R94A;*ble*^R+^ contained a mutated CxrA in which R94 was mutated to alanine. (**H**) Effects of R94 on the CxrA_Δ1–16_ interacting with itself and full-length CxrA analyzed using the yeast two-hybrid system. In panels B–G, the uppercase and lowercase letters indicate *p* < 0.01 and *p* < 0.05, respectively. Different letters indicate significant differences accessed by one-way ANOVA. Each experiment was performed as three biological replicates.

To identify the essential domain in CxrA, DNA sequences encoding a series of truncated CxrA peptides were introduced into the locus of gene *pepA* (*POX*_*d05452*) encoding an aspartic protease [12] in the *P*. *oxalicum* mutant Δ*cxrA*;*G418*^R+^, to generate the corresponding mutants, i.e., Δ61–733;*ble*^R+^, Δ207–733;*ble*^R+^, Δ592–733;*ble*^R+^, Δ1–16;*ble*^R+^, Δ1–60;*ble*^R+^ and Δ1–60&207–733;*ble*^R+^ (Fig 1A), and confirmed by PCR (S1 Fig) with specific primers (S1 Table). In the previous work, the mutant Δ*ku70*Δ*pepA*;*G418*^R+^ showed the same production of cellulase and xylanase relative to the Δ*ku70*;*hph*^R+^, as well as fungal growth on potato dextrose agar (PDA), suggesting that the *pepA* is not involved in the production of cellulase and xylanase, thereby being suitable for gene replacement by expression cassette. This also meant that the Δ*ku70*;*hph*^R+^ can represent the Δ*ku70*Δ*pepA*;*G418*^R+^ at least regarding the production of cellulase and xylanase [12]. When cultured in medium containing Avicel for 2–4 days after transfer from glucose, mutants Δ61–733;*ble*^R+^, Δ1–60;*ble*^R+^ and Δ1–60&207–733;*ble*^R+^ produced cellulase and xylanase at similar levels to Δ*cxrA*;*G418*^R+^, whereas production by mutant Δ592–733;*ble*^R+^ was comparable to that of the complementation strain C*cxrA*;*ble*^R+^. Mutant Δ207–733;*ble*^R+^ exhibited 25.8–26.4% reduced cellulase and xylanase production after 4 days compared with C*cxrA*;*ble*^R+^ (Fig 1B–1D). These results indicated that residues 1–591 of CxrA act like the wild-type CxrA for biosynthesis of cellulase and xylanase in *P*. *oxalicum*, whereas residues 1–206 were sufficient to obtain almost wild type-level CxrA activity (~75%).

Mutant Δ1–16;*ble*^R+^ produced 18.7%–46.1% more cellulase and xylanase than C*cxrA*;*ble*^R+^, suggesting that the oligopeptide CxrA_Δ17–733_ suppressed production of cellulase and xylanase, especially on day 4 (Fig 1B–1D).

The morphological phenotypes of the various *P*. *oxalicum* mutants on agar plates containing various carbon sources were examined. The results indicated that the tested strains exhibited more or less alteration as compared as the complementation strain C*cxrA*;*ble*^R+^. For example, mutant Δ1–60;*ble*^R+^ and Δ1–60&207–733;*ble*^R+^ showed different size and color on PDA plates. In addition, unlike the C*cxrA*;*ble*^R+^, Δ61–733;*ble*^R+^, Δ592–733;*ble*^R+^ and Δ1–16;*ble*^R+^ had less growth on Avicel (S2 Fig).

### Methylation of arginine (R) 94 modulates the biosynthesis of cellulase and xylanase and self-interaction of CxrA_Δ1–60_

To determine whether post-translational modification of CxrA occurred under Avicel induction, the overexpression strain O*cxrA-his*;*G418*^R+^, in which CxrA was tagged with 6×His at the N-terminus, was cultured for 24 h in the presence of Avicel, and total intracellular proteins were extracted. To construct the overexpression strain O*cxrA-his*;*G418*^R+^, as shown in S3A Fig, an overexpression cassette comprised of the coding sequence of *cxrA* with its own promoter, the G418 (antibiotic) resistance gene and both upstream and downstream flanking sequences of gene *pepA* encoding an aspartic protease [12], was introduced into the background strain Δ*ku70*;*hph*^R+^ via homologous recombination. In the O*cxrA-his*;*G418*^R+^, there are two copies of *cxrA* under their native promoters-one at the native locus and the other at the *pepA* locus (S3A Fig). The obtained transformants of O*cxrA-his*;*G418*^R+^ were confirmed by PCR (S3B Fig) with specific primers (S1 Table).

Furthermore, the production of cellulase and xylanase by the overexpression strain O*cxrA-his*;*G418*^R+^ and background strain Δ*ku70*;*hph*^R+^ were compared when cultured in Avicel medium for 2–4 days after transfer from glucose. O*cxrA-his*;*G418*^R+^ showed increased production of all tested cellulases and xylanases by 28.5–68.5% (S3C–S3E Fig) compared with Δ*ku70*;*hph*^R+^. The expression of *cxrA* in O*cxrA-his*;*G418*^R+^ on Avicel was also determined; as expected, the transcriptional abundance of *cxrA* significantly increased in O*cxrA-his*;*G418*^R+^ over that in Δ*ku70*;*hph*^R+^, under Avicel induction for 24 h (S3F Fig). This indicates that *cxrA* was functionally overexpressed, thereby promoting the secretion of cellulase and xylanase on Avicel.

Immunoprecipitation-mass spectrometry was employed to investigate the post-translational modification of CxrA and to detect modifications by acetylation at lysine (K) 30, methylation at R94 and R453, and phosphorylation at threonine (T) 443, T449, T456 and T457 (S4 Fig). As discussed above, the key peptide CxrA_Δ207–733_ contained the acetylated K30 and methylated R94. K30 is required for binding of CxrA to the promoter region of target genes, such as the cellobiohydrolase gene *cbh1*, through its DNA-binding domain CxrA_Δ1–16Δ59–733_ [4]. Therefore, the effects of R94 methylation were investigated on the production of cellulase and xylanase in *P*. *oxalicum*.

The mutant R94A;*ble*^R+^ was constructed, in which the R was changed to alanine (A), and then confirmed by PCR (S1E Fig). When cultured in Avicel medium for 2−4 days after transfer from glucose, R94A;*ble*^R+^ had comparable cellulase and xylanase production to that of Δ*cxrA*;*G418*^R+^, and considerably lower than both the background strain Δ*ku70*;*hph*^R+^ and complementation strain C*cxrA*;*ble*^R+^ (Fig 1E–1G).

To elucidate the function of R94 in the self-interaction of CxrA, yeast two-hybrid (Y2H) analysis was employed. Autoactivation experiments indicated that the full-length CxrA caused autoactivation in *Saccharomyces cerevisiae* (S5 Fig), whereas the peptide CxrA_Δ1–60_ could not (S6 Fig). Therefore, CxrA_Δ1–60_ was used as the bait for the Y2H assay, finding that CxrA_Δ1–60_ interacted with the whole CxrA and itself, whereas the mutated CxrA_Δ1-60_^R94A^ lost the interaction ability (Fig 1H), suggesting that R94 is required for the self-interaction of CxrA_Δ1–60_. Nevertheless, it is possible that interaction of full-length CxrA and itself may be different from the results obtained using the nonfunctional truncation mutant CxrA_Δ1–60_ that may affect protein fold.

### Protein arginine N-methyltransferases PRMT2 and PRMT3 modulate the production of cellulase and xylanase in *P*. *oxalicum*

To search for protein arginine N-methyltransferases that are able to methylate R94 of CxrA, the genomic database of *P*. *oxalicum* strain HP7-1 was screened, finding four annotated protein arginine N-methyltransferases, POX_b03080, POX_d05270, POX_f08428 and POX_e06662 [13]. Of these, three candidates were successfully deleted in *P*. *oxalicum* mutant Δ*ku70*;*hph*^R+^, to generate mutants Δ*POX_b03080*;*G418*^R+^, Δ*POX_d05270*;*G418*^R+^ and Δ*POX_e06662*;*G418*^R+^ (S7 Fig). In addition, an overexpression strain, O*POX_f08428*;*G418*^R+^, was constructed by replacing a protease gene *pepA* [12] in the background strain Δ*ku70*;*hph*^R+^ (S7 Fig), in which the transcription of the *POX_f08428*, was controlled by its own promoter. In the O*POX0_f08428*;*G418*^R+^, there are two copies of *POX_f08428* under their native promoters-one at the native locus and the other at the *pepA* locus (S7A Fig).

**Fig 2:**
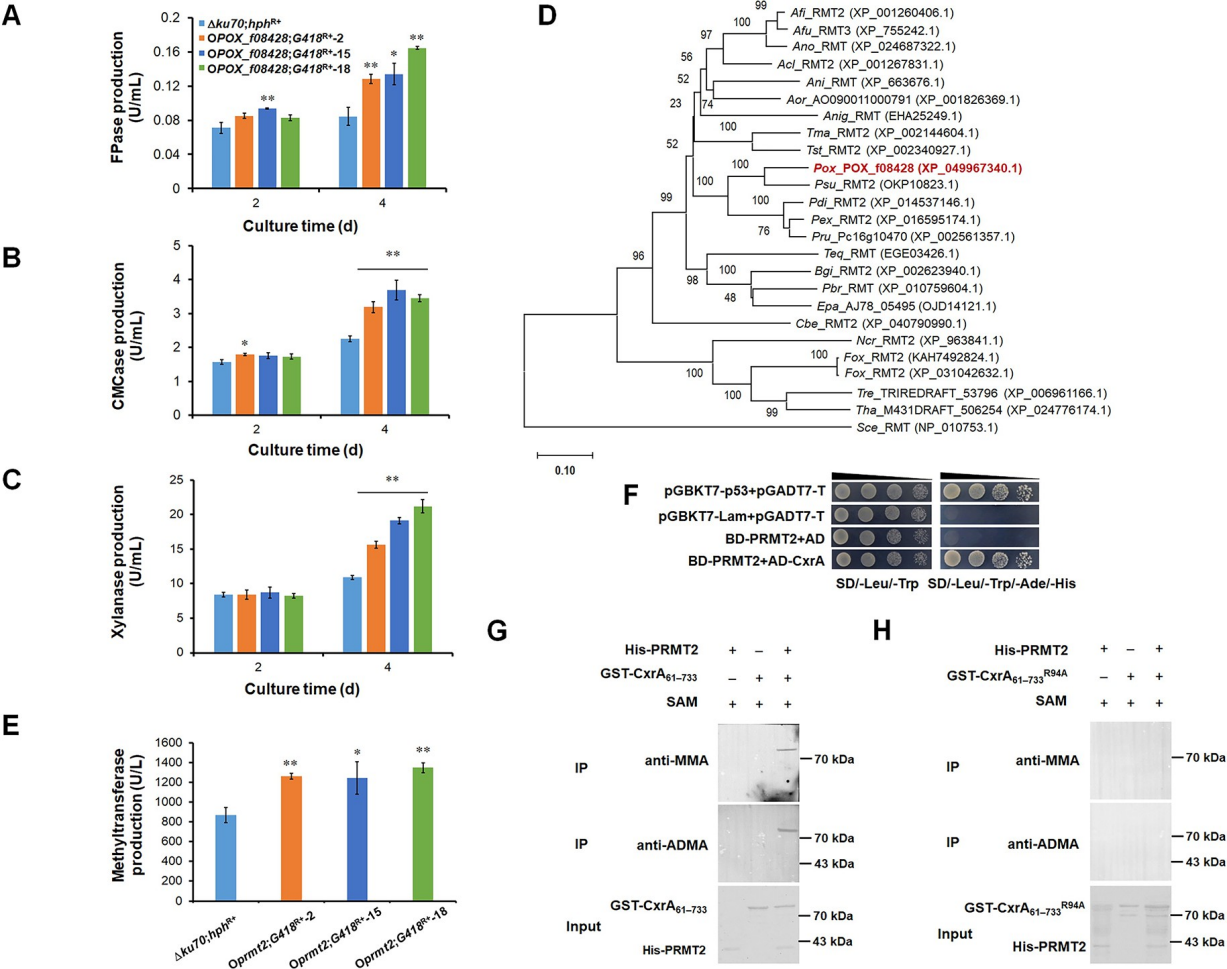
Functional and sequence analysis of arginine N-methyltransferase PRMT2 (POX_f08428) from *P*. *oxalicum*. (**A**) Filter paper cellulase (FPase) production. (**B**) Carboxymethyl cellulase (CMCase) production. (**C**) Xylanase production. (**D**) Phylogenetic analysis of PRMT2. (**E**) Methyltransferase production. (**F**) Yeast two-hybrid assay of PRMT2 and CxrA interaction. (**G**) *In vitro* methylation assay of CxrA_Δ1–60_. (**H**) *In vitro* methylation assay of CxrA_Δ1-60_^R94A^. anti-MMA: mono methyl arginine antibody; anti-ADMA: asymmetric dimethyl arginine antibody; SAM: S-adenosyl-methionine. “+” and “−” indicate the presence or absence of the test protein. In panels A-C, and E, data values indicate means ± standard deviation. **p <0.01 and *p <0.05 indicate significant differences between the mutant and Δ*ku70*, calculated by Student’s *t*-test. In panel D, the phylogenetic trees were constructed by MEGA version 11, using the neighbor-joining method and Poisson model. Bootstrap values shown on the branches were calculated with 1000 replicates. *Afi*: *Aspergillus fischeri*; *Afu*: *Aspergillus fumigatus*; *Ano*: *Aspergillus novofumigatus*; *Acl*: *Aspergillus clavatus*; *Ani*: *Aspergillus nidulans*; *Aor*: *Aspergillus oryzae*; *Anig*: *Aspergillus niger*; *Tma*: *Talaromyces marneffei*; *Tst*: *Talaromyces stipitatus*; *Pox*: *P*. *oxalicum*; *Psu*: *Penicillium subrubescens*; *Pdi*: *Penicillium digitatum*; *Pex*: *Penicillium expansum*; *Pru*: *Penicillium rubens Wisconsin*; *Teq*: *Trichophyton equinum*; *Bgi*: *Blastomyces gilchristii*; *Pbr*: *Paracoccidioides brasiliensis*; *Epa*: *Emergomyces pasteurianus*; *Cbe*: *Cucurbitaria berberidis*; *Ncr*: *Neurospora crassa*; *Fox*: *Fusarium oxysporum*; *Tre*: *Trichoderma reesei*; *Tha*: *Trichoderma harzianum*; *Sce*: *Saccharomyces cerevisiae*.

**Fig 3:**
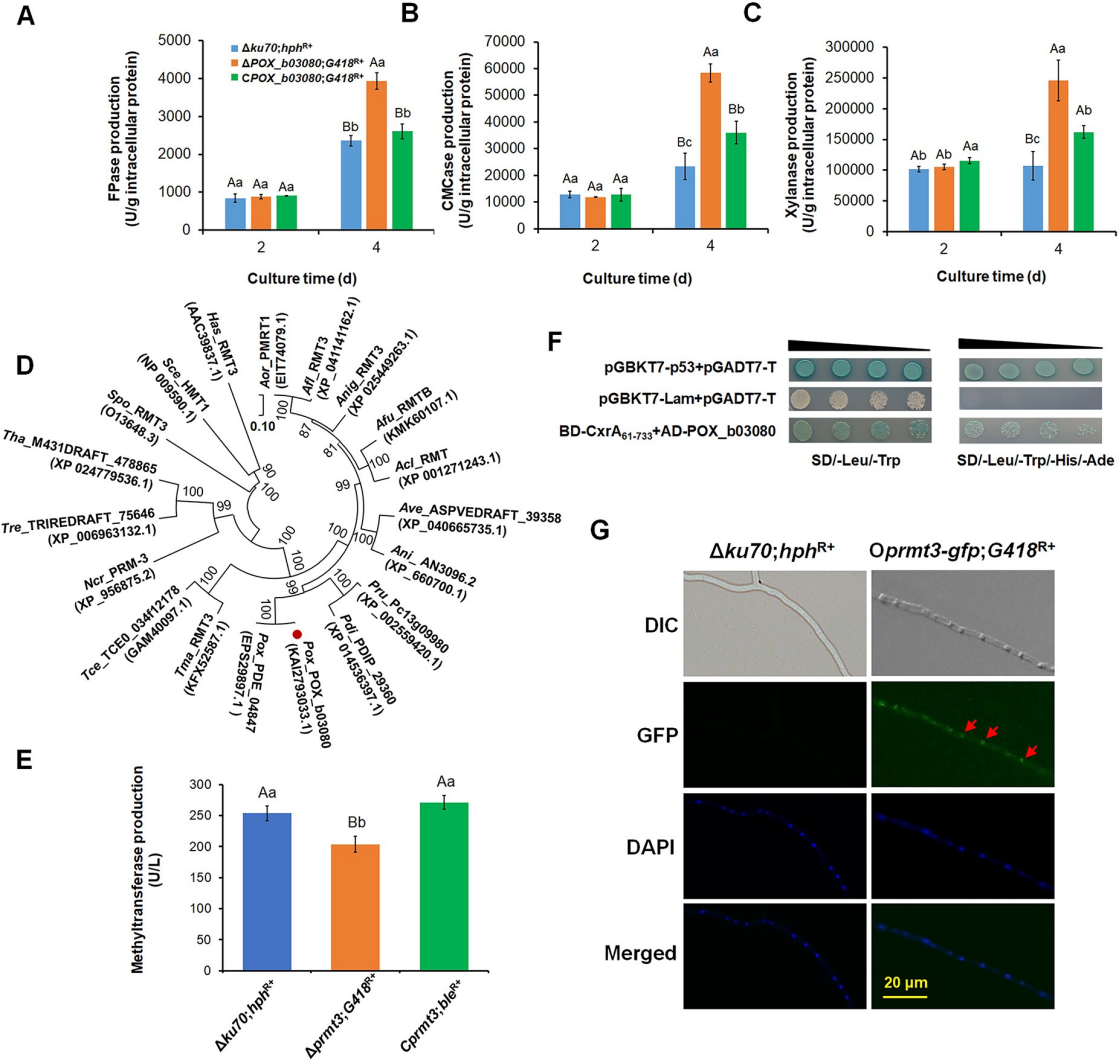
Functional and sequence analysis of arginine N-methyltransferase PRMT3 (POX_d03080) from *P*. *oxalicum*. (**A**) Filter paper cellulase (FPase) production. (**B**) Carboxymethyl cellulase (CMCase) production. (**C**) Xylanase production. (**D**) Evolutionary analysis of PRMT3. (**E**) Methyltransferase production. (**F**) Yeast two-hybrid assay of PRMT3 and CxrA_Δ1–60_ interaction. (**G**) Subcellular localization of PRMT3 in *P*. *oxalicum*. In panels A-C, and E, data values indicate means ± standard deviation. The uppercase and lowercase letters indicate *p* < 0.01 and *p* < 0.05, respectively. Different letters indicate significant differences calculated by one-way ANOVA. In panel D, the phylogenetic trees were constructed by MEGA version 11, using the neighbor-joining method and Poisson model. Bootstrap values shown on the branches were calculated with 1000 replicates. *Afl*: *Aspergillus flavus*; *Afu*: *Aspergillus fumigatus*; *Acl*: *Aspergillus clavatus*; *Ani*: *Aspergillus nidulans*; *Aor*: *Aspergillus oryzae*; *Anig*: *Aspergillus niger*; *Ave*: *Aspergillus versicolor*; *Tma*: *Talaromyces marneffei*; *Pox*: *P*. *oxalicum*; *Pdi*: *Penicillium digitatum*; *Pru*: *Penicillium rubens Wisconsin*; *Ncr*: *Neurospora crassa*; *Tce*: *Talaromyces cellulolyticus*; *Tre*: *Trichoderma reesei*; *Tha*: *Trichoderma harzianum*; *Sce*: *Saccharomyces cerevisiae*; *Spo*: *Schizosaccharomyces pombe*; *Has*: *Homo sapiens*. In panel G, red arrows represent GFP-PRMT3. The expression of *prmt3-gfp* was driven by its own promotor, P*prmt3*, in the overexpression strain O*prmt3-gfp*;*G418*^R+^. DAPI: 4,6-Diamidino-2-phenylindole. GFP: Green fluorescent protein. Scale bar = 20 μm.

When cultured on Avicel medium for 2–4 days after transfer from glucose, only O*POX_f08428*;*G418*^R+^ and Δ*POX_b03080*;*G418*^R+^ exhibited changes in cellulase and/or xylanase production (Figs 2, 3 and S8). For instance, compared with Δ*ku70*;*hph*^R+^, cellulase and xylanase production by O*POX_f08428*;*G418*^R+^ increased by 41.4–95.1% at Day 4 (Fig 2A–2C) and that of Δ*POX_b03080*;*G418*^R+^ increased by 67.0–149.7% (Fig 3A–3C). Furthermore, the expression of *POX_f08428* under Avicel induction for 48 h, significantly increased (S9 Fig). The cellulase and xylanase production of complementation strain C*POX_b03080*;*ble*^R+^ was restored almost to the levels of Δ*ku70*;*hph*^R+^ (Fig 3A–3C). This suggests that POX_f08428 and POX_b03080 positively and negatively modulated the production of cellulase and xylanase in *P*. *oxalicum*, respectively, so the two proteins POX_f08428 and POX_b03080 were selected for further study.

POX_f08428 contained 429 amino acid residues, and an ankyrin repeat domain (IPR002110, residues 61−109). POX_f08428 shared 100%, 66.67% and 34.83% identity, respectively, with PDE_04339 (EPS29390.1) from *P*. *oxalicum* strain 114–2, arginine N-methyltransferase 2 (XP_755242.1) from *Aspergillus fumigatus* strain Af293 and protein-arginine N5-methyltransferase (NP_010753.1) from *S*. *cerevisiae* S288C. POX_f08428 is conserved in eukaryotes and most closely related to its homologs in *Aspergillus* (Fig 2D). For convenience, POX_ f08428 was renamed PRMT2.

POX_b03080 contained 546 amino acid residues and a methyltransfer_25 domain (IPR041698, residues 249 to 346). POX_b03080 shared 99.63%, 40.16% and 36.28% identity, respectively, with PDE_04847 (EPS29897.1) from *P*. *oxalicum* strain 114–2, ribosomal protein arginine N-methyltransferase Rmt3 (NP_595572.1) from *S*. *pombe* 972h^-^ and protein arginine N-methyltransferase 3 (AAC39837.1) from *H*. *sapiens*. POX_b03080 was conserved in eukaryotes and most closely related to its homologs in *Aspergillus* (Fig 3D). For convenience, POX_b03080 was renamed PRMT3.

To investigate the contribution of PRMT2 and PRMT3 to methyltransferase activity in *P*. *oxalicum*, methyltransferase production by overexpression strain O*prmt2*;*G418*^R+^ and mutant Δ*prmt3*;*G418*^R+^ was measured after culture in Avicel medium for 24 h. Methyltransferase production by O*prmt2*;*G418*^R+^ significantly increased (Fig 2E), whereas that of Δ*prmt3*;*G418*^R+^ significantly decreased, compared with Δ*ku70*;*hph*^R+^ and C*prmt3*;*ble*^R+^, and there was no significant difference between Δ*ku70*;*hph*^R+^ and C*prmt3*;*ble*^R+^ (Fig 3E), implying that PRMT2 and PRMT3 have methyltransferase activity.

### PRMT2 is responsible for R94-methylation of CxrA_Δ1–60_

To determin which of PRMT2 and PRMT3 responsible for methylation of R94 in CxrA, the Y2H assay was firstly employed. The results indicated that Y2HGold cells carrying BD-PRMT2 and AD-CxrA (Figs 2F and S10A), as well as BD-CxrA_Δ1–60_ and AD-PRMT3, grew well on SD/-Leu/-Trp/-His/-Ade agar plates (Fig 3F), indicating that the PRMT2 and PRMT3 interact with CxrA and CxrA_Δ1–60_, respectively. However, as unexpected, yeast cells carrying BD-PRMT3 and AD-CxrA were unable to growth on SD/-Leu/-Trp/-His/-Ade agar plates, suggesting that PRMT3 could not interact with the full-length CxrA (S10B Fig). Therefore, this difference maybe result from the use of a nonfunctional CxrA_Δ1–60_ lacking the DNA binding domain. CxrA_Δ1–60_ may affect protein folding.

Furthermore, *in vitro* methylation experiments were performed. As unexpected, DNA sequence encoding a full-length CxrA fused with a glutathione-S-transferase (GST) tag failed to be recombinantly expressed in *E*. *coli* BL21. Therefore, the DNA fragments encoding CxrA_Δ1–60_ fused with a GST tag had to be expressed in *E*. *coli* BL21, as well as PRMT2/PRMT3 tagged with His, then the recombinant GST-CxrA_Δ1–60_ and His-PRMT2 or His-PRMT3 were purified. After reaction of GST-CxrA_Δ1–60_ and His-PRMT3 or His-PRMT2, with S-adenosyl-L-methionine as methyl donor, Western blotting was used to detect methylated CxrA_Δ1–60_ using specific antibodies including anti-mono methyl arginine, anti-symmetric di-methyl arginine, and anti-asymmetric di-methyl arginine. Bands corresponding to methylated GST-CxrA_Δ1–60_ appeared when recombinant His-PRMT2 and GST-CxrA_Δ1–60_ were treated with antibodies anti-mono methyl arginine and anti-asymmetric di-methyl arginine (Fig 2G), respectively, whereas no band was found for the mutated CxrA_Δ1-60_^R94A^ (Fig 2H), showing that PRMT2 both mono-methylated and asymmetrically di-methylated CxrA_Δ1–60_ at R94.

In contrast, the band corresponding to methylated GST-CxrA_Δ1–60_ did not appear when recombinant His-PRMT3 and GST-CxrA_Δ1–60_ were treated with specific antibodies (S11 Fig), indicating that PRMT3 is not responsible for CxrA_Δ1–60_ methylation. Nevertheless, it is possible that *in vitro* methylation modification of the full-length CxrA by PRMT2 or PRMT3 may be different from the results obtained using the nonfunctional truncation mutant CxrA_Δ1–60_ that may affect protein fold.

### PRMT3 appears to be a nuclear arginine N-methyltransferase

To investigate the subcellular localization of PRMT3 in *P*. *oxalicum* hyphae, the overexpression strain O*prmt3*-*gfp*;*G418*^R+^, carrying a GFP reporter was constructed, in which the fused gene *prmt3-gfp* was controlled by its own promoter, P*prmt3* (S7 Fig). In the O*prmt3-gfp*;*G418*^R+^, there are two copies of *prmt3* under their native promoters-one at the native locus and the other at the *pepA* locus (S7D Fig). After culture in Avicel medium for 24 h, the hyphae of O*prmt3*-*gfp*;*G418*^R+^ and background strain Δ*ku70*;*hph*^R+^ were observed under a fluorescence microscope. The green fluorescence of the fusion protein, PRMT3-GFP, in O*prmt3*-*gfp*;*G418*^R+^ appeared to overlap with blue fluorescence signals from 4,6-diamidino-2-phenylindole (DAPI), which specifically stains the nucleus, whereas there was no green fluorescence from Δ*ku70*;*hph*^R+^ (Fig 3G).

### PRMT3 mediates the expression of genes related to cellulase and xylanase biosynthesis by *P*. *oxalicum*

To elucidate the effects of PRMT3 on the expression of genes related to cellulase and xylanase production by *P*. *oxalicum*, RNA-sequencing and a real-time quantitative reverse transcription PCR (RT-qPCR) assay were employed. Both Δ*ku70*;*hph*^R+^ and Δ*prmt3*;*G418*^R+^ were cultured in Avicel medium for 24 h, after transfer from glucose, and their total RNA was extracted for RNA-sequencing. The sequencing data from three biological replicates were analyzed statistically and generated a high Pearson correlation coefficient (> 0.97) (S12 Fig), suggesting that the transcriptomic data were suitable for the subsequent analysis.

**Fig 4:**
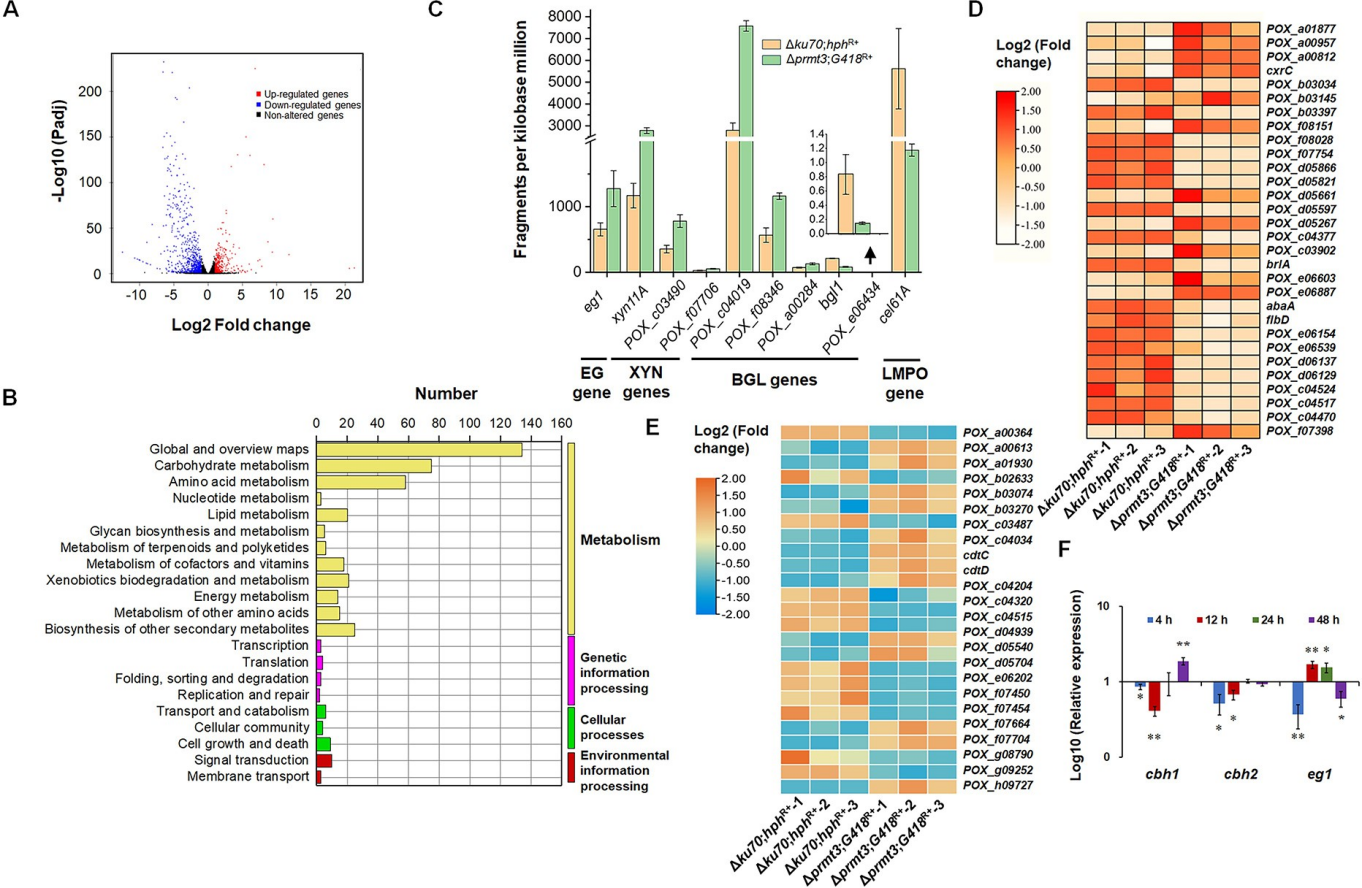
Comparative analyses of transcriptomes from *P*. *oxalicum* mutant Δ*prmt3*;*G418*^R+^ and background strain Δ*ku70*;*hph*^R+^ (A-E) and real-time reverse transcription quantitative PCR (RT-qPCR) confirmation (F). (**A**) Volcano plot of differentially expressed genes (DEGs). DEGs were selected with thresholds of |log2 fold change| > 1.0 and *p*-value < 0.05. (**B**) KEGG annotations of the DEGs modulated by PRMT3. (**C**) DEGs encoding cellulases and xylanases. (**D**) DEGs encoding putative transcription factors. (**E**) DEGs encoding putative sugar transporters. In panels D and E, the three columns of data corresponding to each fungal strain represent the three biological replicates for each strain. (**F**) The transcriptional levels of genes encoding major cellulases including two cellobiohydrolase genes *cbh1* and *cbh2*, and an endo-β-1,4-glucanase gene *eg1* in the *P*. *oxalicum* mutant Δ*prmt3*;*G418*^R+^ relative to the background strain Δ*ku70*;*hph*^R+^. In panel F, the *P*. *oxalicum* strains were cultured for 4–48 h in the presence of Avicel. Gene expression in Δ*prmt3*;*G418*^R+^ was normalized to the level of Δ*ku70*;*hph*^R+^. Data points show mean ± standard deviation. **p <0.01 and *p <0.05 indicate significant differences between Δ*prmt3*;*G418*^R+^ and Δ*ku70*;*hph*^R+^, calculated by Student’s *t*-test.

With thresholds of |log2 fold change| > 1 and false discovery rate (FDR) < 0.05, there were 997 differentially expressed genes (DEGs) detected in Δ*prmt3*;*G418*^R+^ compared with Δ*ku70*;*hph*^R+^, consisting of 321 up-regulated and 676 down-regulated genes (Fig 4A and S1 Dataset). The Kyoto Encyclopedia of Genes and Genomes (KEGG) annotation showed that these DEGs were mainly involved in metabolism, especially carbohydrate metabolism (75 genes) and amino acid metabolism (58 genes) (Fig 4B).

Among the 997 DEGs, nine cellulase/xylanase genes were found, namely one endo-β-1,4-glucanase gene (*POX_d04883/eg1*), five β-glucosidase genes (*bgl*s, *POX_a00284*, *POX_c04019*, *POX_e06772/bgl1*, *POX_e06434* and *POX_f08346*) and three endo-β-1,4-xylanase genes (*POX_a01871*/*xyn11A*, *POX_c03490* and *POX_f07706*). Of these, *POX_b02315/cel61A*, *POX_e06772/bgl1* and *POX_e06434* were down-regulated (-2.4 < log2 fold change < -1.4), and the others were up-regulated (1.0 < log2 fold change < 1.6) in Δ*prmt3*;*G418*^R+^ compared with Δ*ku70*;*hph*^R+^ (Fig 4C).

The effects of PRMT3 on the expression of genes encoding putative TFs was then investigated by comparative analysis of transcriptomes, which identified 30 TF-encoding DEGs. The transcript abundances of 12 of them increased by 2.03–52.6-fold in Δ*prmt3*;*G418*^R+^, whereas the other 18 genes decreased by 1.2–499-fold. Most of these TFs contained zinc-finger domains (i.e., six C2H2-type, 11 Zn2Cys6-type and one CCHC-type). Notably, expression of a key transcription repressor gene, *cxrC* in Δ*prmt3*;*G418*^R+^ increased 2.46-fold, whereas transcripts of sporulation-regulated *abaA*, *brlA* and *flbD* decreased by 59.4–95.9% (Fig 4D).

In addition, 25 DEGs encoding predicted sugar transporters were found, of which 13 were up-regulated (1.0 < log_2_ fold change < 2.9) and 12 down-regulated (-4.2 < log2 fold change < -1.3) in Δ*prmt3*;*G418*^R+^. Of these, two major cellodextrin transporter genes, *cdtC* and *cdtD* were up-regulated 2.14- and 2.30-fold, respectively (Fig 4E).

Three cellulase genes, *cbh1*, *cbh2*, and *eg1* were subjected to RT-qPCR analysis of expression variation with induction duration and confirmation of RNA-sequencing data. The expression of *eg1* after 24 h of induction was up-regulated in Δ*prmt3*;*G418*^R+^ compared with Δ*ku70*;*hph*^R+^, whereas *cbh1* and *cbh2* showed no significant change, in agreement with the RNA-sequencing results. Transcriptional abundances of all three genes decreased up to 12 h, except for *eg1* at 12 h, which markedly increased. At 48 h, the expression of *cbh1* was up-regulated, whereas *eg1* was down-regulated. The transcriptional level of *cbh2* did not change (Fig 4F).

### PRMT3 was required for positive regulation of cellulase and xylanase production by *cxrA*

As described above, PRMT3 was unable to methylate CxrA, but deletion of *prmt3* from *P*. *oxalicum* increased cellulase and xylanase production by 67.0–149.7%, compared with the background strain Δ*ku70*;*hph*^R+^ after 4 days of Avicel induction (Fig 3). To investigate the effects of PRMT3 on CxrA action, the *cxrA*-overexpression strain O*cxrA-gfp*;*G418*^R+^ and mutant Δ*prmt3*;*cxrA*-*gfp*; *G418*^R+^;*ble*^R+^ was sequentially constructed and confirmed by PCR (S7 Fig). In O*cxrA*-*gfp*;*G418*^R+^, the fusion gene *cxrA-gfp* was controlled by the predicted natural promoter of *cxrA*. In the O*cxrA-gfp*;*G418*^R+^ and Δ*prmt3*;*cxrA*-*gfp*;*G418*^R+^;*ble*^R+^, there are two copies of *cxrA* under their native promoters- one at the native locus and the other at the *pepA* locus (S7E and S7F Fig). RT-qPCR confirmed that the expression of *cxrA* in O*cxrA*-*gfp*;*G418*^R+^ significantly increased, compared with that in Δ*ku70*;*hph*^R+^ after 48 h of Avicel induction (S9 Fig).

**Fig 5:**
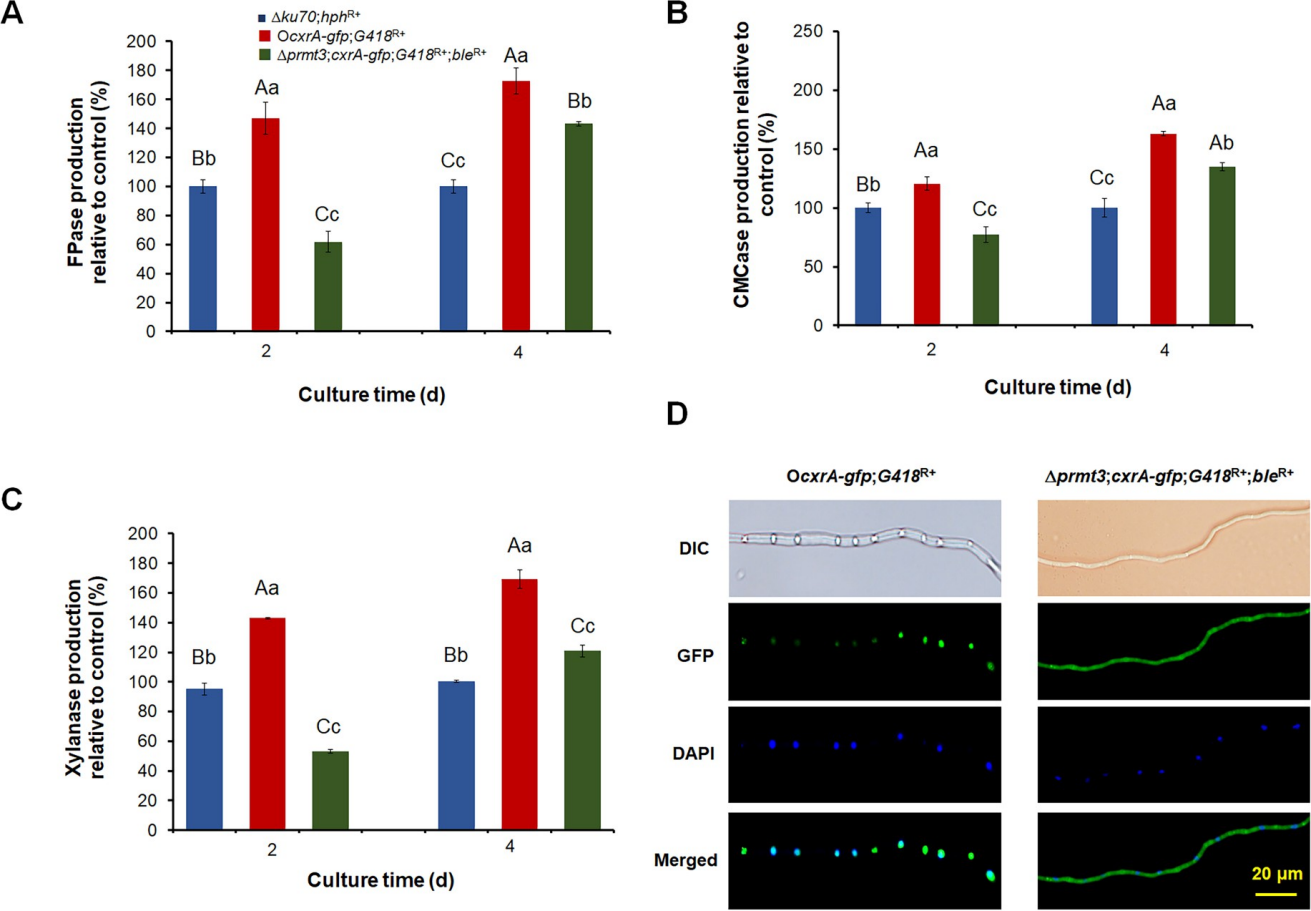
Effects of PRMT3 on the regulatory functions of CxrA. (**A**) Filter paper cellulase (FPase) production. (**B**) Carboxymethyl cellulase (CMCase) production. (**C**) Xylanase production. (**D**) Subcellular localization of CxrA in *P*. *oxalicum*. In panels A-C, the fungal strains were pre-grown in glucose medium for 24 h, then transferred into Avicel medium for 2–4 d. The uppercase and lowercase letters indicate *p* < 0.01 and *p* < 0.05, respectively. Different letters indicate significant differences assessed by one-way ANOVA. All tested samples were normally distributed. In panel D, 4,6-diamidino-2-phenylindole (DAPI) was employed to stain mycelial nuclei. Subcellular localization of CxrA was determined with GFP signals observed by a fluorescence microscope. Scale bar = 20 μm.

Measurement of enzymatic activity revealed that cellulase and xylanase production by Δ*prmt3*;*cxrA*-*gfp*;*G418*^R+^;*ble*^R+^ was 11.3–62.8% lower than that of the overexpression strain O*cxrA*-*gfp*;*G418*^R+^, when cultured in Avicel medium for 2–4 d (Fig 5A–5C), indicating that PRMT3 is required for positive regulation of cellulase and xylanase production by *cxrA*.

### PRMT3 apparently assists the entry of CxrA into the nucleus

To further elucidate the interactions between CxrA and PRMT3, Δ*prmt3*;*cxrA*-*gfp*;*G418*^R+^;*ble*^R+^ and O*cxrA*-*gfp*;*G418*^R+^ were cultured in Avicel medium for 24 h, then their hyphae were collected for microscopic examination. The green fluorescent signals from CxrA-GFP in O*cxrA*-*gfp*;*G418*^R+^ appeared to overlap with the blue fluorescence signals from DAPI, indicating that CxrA was localized in the nucleus, whereas the green fluorescence signals from CxrA-GFP in Δ*prmt3*;*cxrA*-*gfp*;*G418*^R+^;*ble*^R+^ were evenly distributed in the whole hypha. This seems that deletion of *prmt3* partially obstructed nuclear translocation of CxrA protein, in other words, it appears that PRMT3 assists the entry of CxrA into the nucleus (Fig 5D).

### Co-expression analysis of genes regulated by CxrA and PRMT3

Co-expression analysis of genes regulated by PRMT3 and CxrA was performed by RNA-sequencing of Δ*prmt3*;*G418*^R+^ and Δ*cxrA*;*G418*^R+^, compared with Δ*ku70*;*hph*^R+^ cultured in Avicel medium for 24 h, after transfer from glucose. The resulting data from three biological replicates had a high Pearson’s correlation coefficient (> 0.97) (S12 Fig).

With thresholds of |log2 fold change| > 1 and FDR < 0.05, there were 2,552 DEGs in Δ*cxrA*;*G418*^R+^ compared with Δ*ku70*;*hph*^R+^, of which 1,253 were up-regulated and 1,299 down-regulated (S2 Dataset). These included 27 key cellulase and xylanase genes, 128 putative TF-encoding genes and 28 putative sugar transporter-encoding genes (S2 Dataset), in agreement with a previous report [4].

**Fig 6:**
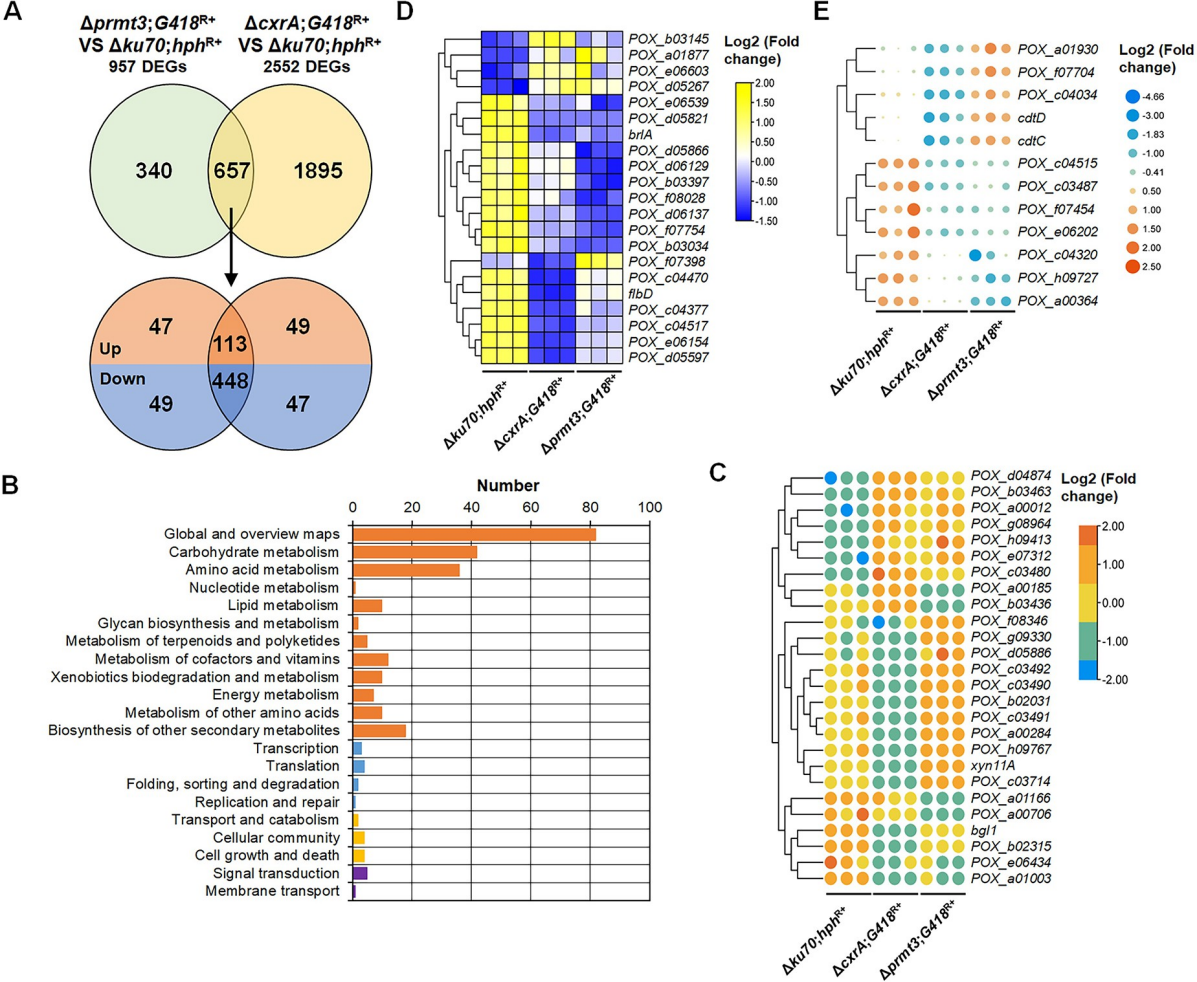
Co-regulation between CxrA and PRMT3 in *P*. *oxalicum*. (**A**) Numbers of differentially expressed genes (DEGs) in regulons between *cxrA* and *prmt3*. DEG regulons were determined by comparison of transcriptomic data from Δ*prmt3*;*G418*^R+^ and Δ*cxrA*;*G418*^R+^ with that of the background strain Δ*ku70*;*hph*^R+^. “Up” and “Down” indicate up- and down-regulation. (**B**) KEGG annotations of the 657 genes co-regulated by *cxrA* and *prmt3*. (**C**) Heatmap illustrating the expression of DEGs encoding plant-cell-wall-degrading enzymes. (**D**) Heatmap showing the expression of DEGs encoding putative transcription factors. (**E**) Heatmap displaying the expression of DEGs encoding sugar transporters. In panels C and D, the three columns of data corresponding to each fungal strain represent the three biological replicates for each strain.

Comparative analysis identified 657 DEGs co-regulated by PRMT3 and CxrA under Avicel induction, of which 113 were up-regulated and 448 down-regulated in Δ*prmt3*;*G418*^R+^ and Δ*cxrA*;*G418*^R+^, relative to Δ*ku70*;*hph*^R+^ (Fig 6A). These co-regulated genes were mainly involved in metabolism, especially carbohydrate and amino acid metabolism (Fig 6B).

The co-regulated DEG set included four BGL genes (*bgl1*, *POX_a00284*, *POX_f08346* and *POX_e06434*) and two xylanase genes (*xyn11A* and *POX_c03490*). The mRNA/transcription levels of these genes were up-regulated in Δ*prmt3*;*G418*^R+^, but down-regulated in Δ*cxrA*;*G418*^R+^, compared with Δ*ku70*;*hph*^R+^, except for *bgl1* and *POX_e06434*, which were down-regulated in both mutants (Fig 6C). The expression of most co-regulated DEGs encoding TFs and sugar transporters decreased in Δ*prmt3*;*G418*^R+^ and Δ*cxrA*;*G418*^R+^, compared with Δ*ku70*;*hph*^R+^ (Fig 6D and 6E), however, the cellodextrin transporter genes *cdtC* and *cdtD* were downregulated in Δ*cxrA*;*G418*^R+^ and upregulated in Δ*prmt3*;*G418*^R+^, compared with Δ*ku70*;*hph*^R+^ (Fig 6E).

Notably, gene *prmt2* was included in the CxrA regulon, rather than in the *prmt3* regulon. In Δ*cxrA*;*G418*^R+^, the expression of *prmt2* increased by 1.2-fold, compared with Δ*ku70*;*hph*^R+^ (S2 Dataset), indicating that CxrA inhibits the expression of *prmt2* on Avicel.

## Discussion

Previous work demonstrated that an Zn2Cys6-type TF, CxrA, promotes the biosynthesis of cellulase and xylanase in *P*. *oxalicum* [2]. From this study, it appears that the N-terminal region, CxrA_Δ207–733_ is essential to the regulatory function of full-length CxrA, containing the DNA-binding domain (CxrA_Δ1–16&Δ59–733_) [4] and the methylation site, R94 under induction conditions. Nevertheless, in general, for many Zn2Cys6-type TFs such as ClrB, the C-terminal region is capable of transcriptional activation and the intermediate region participates in the regulation of TF activity [14]. In addition, the N-terminal CxrA_Δ17–733_ repressed the regulatory action of CxrA by an unknown mechanism.

Moreover, CxrA appeared to interact with the truncation mutant CxrA_Δ1–60_ via Y2H assay, i.e., dimerize, in common with other TFs belonging to the zinc finger family, such as ACE3 [15], CLR-2, XLR-1 [16] and CxrC [5]. The truncation mutant CxrA_Δ1–60_ may affect protein fold, thereby leading to the different results from interaction between the full-length CxrA and itself. However, whether self-interaction of the full-length CxrA occurs actually in *P*. *oxalicum* merits further investigation, as well as whether this interaction is required for CxrA function.

**Fig 7:**
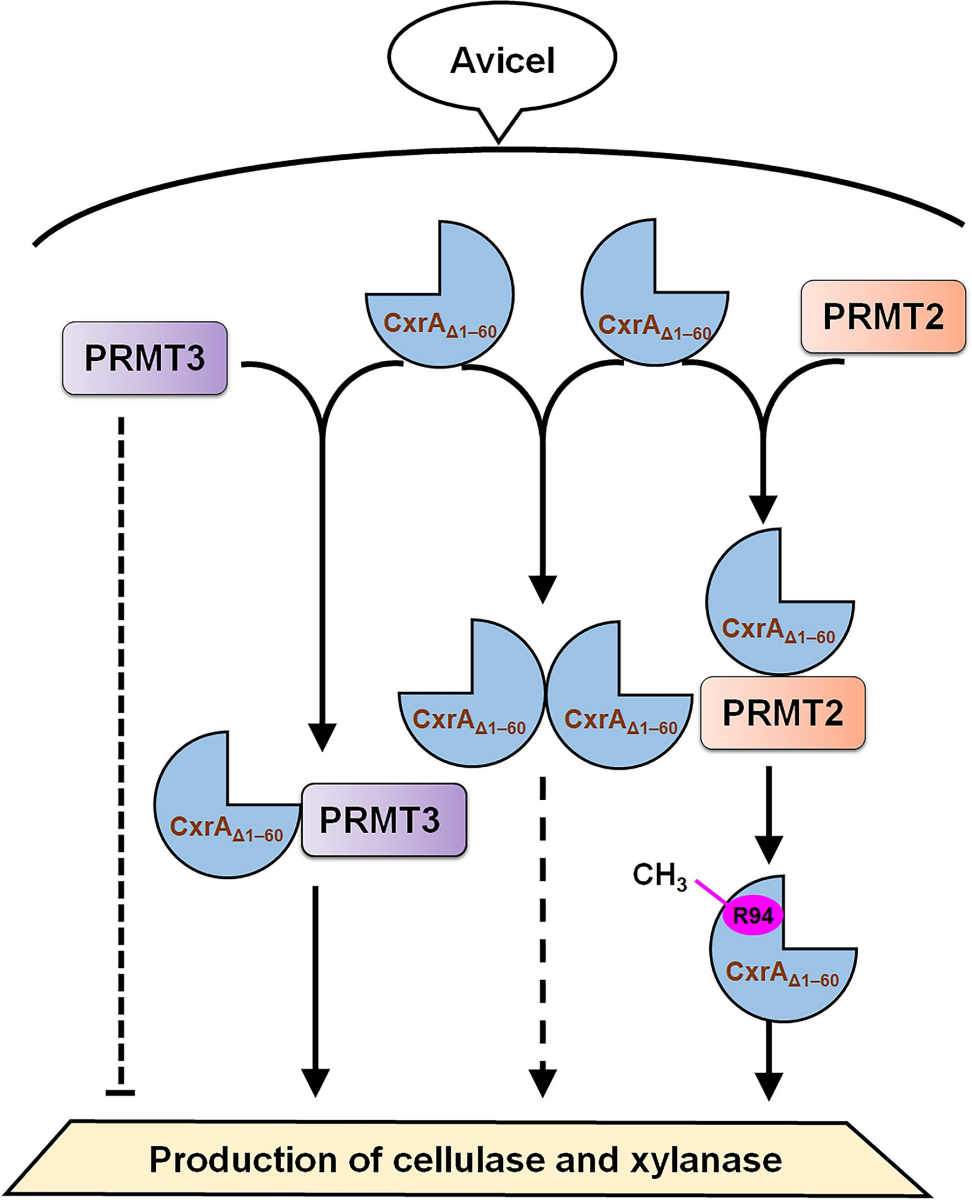
Proposed model of CxrA function in *P*. *oxalicum* grown on Avicel. CxrA_Δ1–60_ is methylated by an arginine N-methyltransferase PRMT2 at R94, and interacts with itself. In addition, the CxrA_Δ1–60_ interacts with another arginine N-methyltransferase, PRMT3, which apparently assists the nuclear translocation of CxrA. PRMT3 down-regulates the production of cellulase and xylanase in wild-type *P*. *oxalicum*, when growing on Avicel, by an unknown mechanism, but PRMT3 is also required for *cxrA*-mediated up-regulation of cellulase and xylanase production by *P*. *oxalicum*. Notably, the role for PRMT3 in regulating the expression of major cellulase and xylanase expression may change over time. The order of occurrence of the interaction between CxrA_Δ1–60_ and PRMT3, CxrA_Δ1–60_ self-interaction/dimerization, and CxrA_Δ1–60_ methylation remains unclear. Nevertheless, it should be noted that the CxrA_Δ1–60_ used for *in vitro* methylation experiments is nonfunctional and lacks the DNA binding domain, and thus, that it is possible that interactions between full-length CxrA and PRMT2 may be different due to changes in protein folding. Lines with arrows display activation, and barred lines show repression. Dashed lines indicate an unclear mechanism.

Post-translational modification generally modulates protein function, especially methylation, phosphorylation, and acetylation. Although the exact function of arginine methylation is still controversial, accumulated evidence indicates that it is involved in many cellular processes, such as transcription activation and repression, protein-protein interaction, DNA repair and pre-mRNA splicing [17,18]. However, these reports mainly focus on humans and other mammals [19–25] and to the best of our knowledge, there is only one previous report on arginine methylation in microorganisms [11]. This study found that R94 in CxrA_Δ1–60_ was methylated by protein arginine N-methyltransferase PRMT2. Nevertheless, it should be noted that the truncated CxrA_Δ1–60_ used for *in vitro* methylation experiments is nonfunctional and lacks the DNA binding domain, and thus, that it is possible that interactions between full-length CxrA and PRMT2 may be different due to changes in protein folding, etc. Moreover, R94 was required for the activation by CxrA of cellulase and xylanase biosynthesis (Fig 7), as well as the interaction between CxrA_Δ1–60_ and itself, which might be due to the methylation of R94. However, it did not exclude the possibility that the exchange of the amino acid maybe simply lead to misfolding, which requires further observation by either CD-spectroscopy or HDX-MS. How methylation modification influences the regulatory roles of CxrA also remains unclear.

Homologous alignment indicated that PRMT2 shared a low identity (34.83%) with type IV PRMT, RMT2 from yeast [26]. RMT2 catalyzes the formation of *δ*-*N*^G^ monomethyl arginine [27]. Notably, *in vitro* methylation experiments confirmed that PRMT2 could catalyze the biosynthesis of the *ω*-*N*^G^ monomethyl arginine and asymmetric *ω-N*^*G*^, *N*^G^-dimethylarginine [6]; this activity would normally be classified as a type I PRMT, so further study will be needed to clarify the correct classification of PRMT2.

It should be noted that the it was not possible to generate the deletion mutant Δ*prmt2*;*G418*^R+^, suggesting that PRMT2 is critical to cell-survival and that deletion results in Δ*prmt2*;*G418*^R+^ becoming a non-viable mutant. Overexpression of *prmt2* up-regulated cellulase and xylanase production under Avicel induction, and it is possible that increasing R94 methylation of CxrA strengthens its regulatory effect.

Moreover, another arginine methyltransferase PRMT3 was also identified, which down-regulated cellulase and xylanase production at the late stage of Avicel induction in wild-type *P*. *oxalicum*, whereas PRMT3 was required for up-regulation of cellulase and xylanase production by *cxrA*. Notably, the role for PRMT3 in regulating cellulase and xylanase expression may change over time. In addition, unexpectedly, PRMT3 interacted with CxrA_Δ1–60_ but not with full-length CxrA via Y2H assay, whereas it did not methylate CxrA_Δ1–60_ (Fig 7). In addition, it appears that nuclear translocation of CxrA is facilitated by PRMT3, but that CxrA can also enter the nucleus independently of PRMT3. The different interactions between PRMT2 and the full-length CxrA, and nonfunctional CxrA might be due to changes in protein folding.

PRMT3 is highly conserved in all eukaryotes [17] and is essential to a variety of cellular processes, for examples, PRMT3 influences the relative levels of small ribosomal subunits in yeast, by interaction with Rps2 (40S ribosomal protein S2), but not by methylation [19]; negatively regulates antiviral responses in Zebrafish [20]; stimulates tumorigenesis via controlling c-MYC stabilization in colorectal cancer [21]; represses retinoic acid signaling through interacting with retinal dehydrogenase 1 [22]. This study initially found that PRMT3 influenced the cellulase and xylanase biosynthesis in *P*. *oxalicum* through unknown mechanism.

An important issue arising from the above findings is the apparent paradox that PRMT3 suppresses cellulase and xylanase biosynthesis in the wild-type *P*. *oxalicum*, but it is required for up-regulation of cellulase and xylanase production by *cxrA*. It appears that PRMT3 facilitates the entry of CxrA into the nucleus, repressed the expression of the important transcriptional repressor gene, *cxrC* [5] and dynamically regulated some cellulase and xylanase genes. This implies that PRMT3 repressed cellulase and xylanase production in *P*. *oxalicum* through a complex mechanism. To explain this mystery, the substrates methylated by PRMT3 in *P*. *oxalicum* should be identified. In addition, the regulatory effects of PRMT3 conflict with activation of CxrA, suggesting that genetic engineering involving their genes, to improve the yields of fungal cellulase and xylanase in synthetic biology, will be very challenging until their functions and interactions are much more clearly understood.

Improved understanding of the functions of PRMTs is not only relevant to fungi, but also to human health. PRMT is considered a promising target for inhibition, with great potential for such inhibitors in cancer therapy [18] and treatment of other diseases. For example, the allosteric PRMT3 inhibitor SGC707 effectively reduced the extent of hepatic steatosis (fatty liver) in mice [28]. PRMT inhibition may also be possible by the alternative approach of down-regulating the expression of PRMT-encoding genes. Further research is needed to identify more regulatory genes and to improve understanding of cellulase and xylanase biosynthesis regulation in *P*. *oxalicum*.

## Materials and methods

### Microbial strains, medium and culture conditions

All microbial strains used in this study are shown in Table 1. *E*. *coli* strains DH5α and DE3 were used for plasmid construction and protein heterologous expression, and were cultured in Luria-Bertani medium at 37°C.

**Table 1:** *Penicillium oxalicum* strains used in this study.

*P*. *oxalicum* strains	Description	Genotypes	References
Δ*ku70*;*hph*^R+^	Background strain in which gene *ku70* is replaced by *hph*^R+^	*ku70*^−^; *hph*^R+^	[29]
Δ*ku70*Δ*pepA*; *G418*^R+^	In the Δ*ku70*::*hph*^R+^, gene *pepA* is replaced by *G418*^R+^	*ku70*^−^; *pepA*^−^; *hph*^R+^; *G418*^R+^	[12]
Δ*cxrA*;*G418*^R+^	In the Δ*ku70*::*hph*^R+^, gene *cxrA* is replaced by *G418*^R+^	*ku70*^−^; *cxrA*^−^; *hph*^R+^; *G418*^R+^	[2]
C*cxrA*;*ble*^R+^	In the Δ*cxrA*::*G418*^R+^, the full *cxrA* is integrated at *pepA* locus, with the marker *ble*^R+^	*ku70*^−^; *pepA*^−^; *hph*^R+^; *G418*^R+^; *ble*^R+^	This study
Δ61–733;*ble*^R+^	In the Δ*cxrA*::*G418*^R+^, *cxrA*_Δ61–733_ is integrated at *pepA* locus, with the marker *ble*^R+^	*ku70*^−^; *pepA*^−^; *cxrA*^−^; *cxrA*_Δ61–733_^+^; *hph*^R+^; *G418*^R+^; *ble*^R+^	This study
Δ207–733;*ble*^R+^	In the Δ*cxrA*::*G418*^R+^, *cxrA*_Δ207–733_ is integrated at *pepA* locus, with the marker *ble*^R+^	*ku70*^−^; *pepA*^−^; *cxrA*^−^; *cxrA*_Δ207–733_^+^; *hph*^R+^; *G418*^R+^; *ble*^R+^	This study
Δ592–733;*ble*^R+^	In the Δ*cxrA*::*G418*^R+^, *cxrA*_Δ592–733_ is integrated at *pepA* locus, with the marker *ble*^R+^	*ku70*^−^; *pepA*^−^; *cxrA*^−^; *cxrA*_Δ592–733_^+^; *hph*^R+^; *G418*^R+^; *ble*^R+^	This study
Δ1–16;*ble*^R+^	In the Δ*cxrA*::*G418*^R+^, *cxrA*_Δ1–16_ is integrated at *pepA* locus, with the marker *ble*^R+^	*ku70*^−^; *pepA*^−^; *cxrA*^−^; *cxrA*_Δ1–16_^+^; *hph*^R+^; *G418*^R+^; *ble*^R+^	This study
Δ1–60;*ble*^R+^	In the Δ*cxrA*::*G418*^R+^, *cxrA*_Δ1–60_ is integrated at *pepA* locus, with the marker *ble*^R+^	*ku70*^−^; *pepA*^−^; *cxrA*^−^; *cxrA*_Δ1–60_^+^; *hph*^R+^; *G418*^R+^; *ble*^R+^	This study
Δ1–60&207–733;*ble*^R+^	In the Δ*cxrA*::*G418*^R+^, *cxrA*_Δ1–60&207–733_ is integrated at *pepA* locus, with the marker *ble*^R+^	*ku70*^−^; *pepA*^−^; *cxrA*^−^; *cxrA*_Δ1–60&207–733_^+^; *hph*^R+^; *G418*^R+^; *ble*^R+^	This study
R94A;*ble*^R+^	In the Δ*cxrA*::*G418*^R+^, *cxrA*^R94A^ is integrated at *pepA* locus, with the marker *ble*^R+^	*ku70*^−^; *pepA*^−^; *cxrA*^−^; *cxrA*^R94A+^; *hph*^R+^; *G418*^R+^; *ble*^R+^	This study
Δ*prmt3*;*G418*^R+^	In the Δ*ku70*::*hph*^R+^, gene *prmt3* is replaced by *G418*^R+^	*ku70*^−^; *prmt3*^−^; *hph*^R+^; *G418*^R+^	This study
O*prmt2*;*G418*^R+^	In the Δ*ku70*::*hph*^R+^, gene *prmt2* is integrated at gene *pepA*, with the maker *G418*^R+^	*ku70*^−^; *pepA*^−^; two copies of *prmt2*; *hph*^R+^; *G418*^R+^	This study
C*prmt3*;*ble*^R+^	In the Δ*cxrA*::*G418*^R+^, the full *prmt3* is integrated at *pepA* locus, with the marker *ble*^R+^	*ku70*^−^; *pepA*^−^; *hph*^R+^; *G418*^R+^; *ble*^R+^	This study
O*prmt3*;*G418*^R+^	In the Δ*ku70*::*hph*^R+^, gene *prmt3* is integrated at gene *pepA*, with the maker *G418*^R+^	*ku70*^−^; *pepA*^−^; two copies of *prmt3*; *hph*^R+^; *ble*^R+^	This study
O*cxrA-gfp*;*G418*^R+^	In the Δ*ku70*::*hph*^R+^, the *cxrA*-*gfp* is integrated at gene *pepA*, with the maker *G418*^R+^	*ku70*^−^; two copies of *cxrA*; *gfp*^+^; *hph*^R+^; *G418*^R+^	This study
Δ*prmt3*;*cxrA-gfp*;*G418*^R+^;*ble*^R+^	In the Δ*prmt3*::*G418*^R+^, the *cxrA*-*gfp* is integrated at gene *pepA*, with the maker *ble*^R+^	*ku70*^−^; *prmt3*^−^; *G418*^R+^; two copies of *cxrA*; *gfp*^+^; *hph*^R+^; *ble*^R+^	This study

*S*. *cerevisiae* strains Y2HGold and Y187, used for the Y2H assay, were cultured at 30°C in yeast extract peptone dextrose medium (YPD) containing peptone (20.0 g/L), yeast extract (10.0 g/L), glucose (20.0 g/L) and adenine (4.0 g/L). Synthetic defined medium (SD Base) lacking tryptophan, leucine, histidine, and adenine (SD/–Trp/–Leu/–His/–Ade), with Aureobasidin A (AbA, 100 ng/mL) was used for screening of recombinant yeast.

*P*. *oxalicum* strains, including the background strain Δ*ku70*;*hph*^R+^ [29] and a series of constructed mutants were routinely cultured at 28°C on PDA plates for 4−5 d. The conidia were collected with 0.2% Tween 80 (Sangon Biotech Co., Ltd., Shanghai, China) from the plates, and used for reproduction.

To measure the production of cellulase and xylanase, *P*. *oxalicum* strains were pre-grown in modified minimal medium (MMM; g/L, (NH4)_2_SO_4_ 4.0, CaCl_2_ 0.6, KH_2_PO_4_ 4.0, MgSO_4_·7H_2_O 0.6, FeSO_4_·7H_2_O 0.25, MnSO_4_·H_2_O 0.08, ZnCl_2_ 0.085, CoCl_2_ 0.1, 2 ml/L Tween 80, pH 5.5) containing 1% glucose as the sole carbon source for 24 h, then transferred into MMM containing 2% Avicel as the carbon source [5] for 2–4 d.

*P*. *oxalicum* strains were cultured on PDA and MMM agar plates containing 1% glucose, 2% CMC, or 2% Avicel as carbon source, at 28°C for 4 d, for observation of colony phenotype.

For RNA-sequencing and RT-qPCR assays, *P*. *oxalicum* strains were cultured at 28°C for 4–48 h, after a transfer from glucose (1%) medium, as described above.

### Yeast two-hybrid (Y2H) assay

The Y2H assay was performed with the Matchmaker Gold Yeast Two-Hybrid System, following the manufacturer’s instructions (TaKaRa, Dalian, China). The DNA fragments encoding CxrA_Δ1–60_, CxrA_Δ1-60_^R94A^ and PRMT2/3 used as the baits were amplified from the genomic DNA of HP7-1 and cloned into the plasmid pGBKT7 at *Eco*RI/*Bam*HI sites, respectively, resulting in the recombinant pGBKT7-*cxrA*_Δ1–60_, pGBKT7-*cxrA*_Δ1-60_^R94A^, pGBKT7-*prmt2* and pGBKT7-*prmt3*. The DNA fragments encoding PRMT2/3 and CxrA used as the preys, from *P*. *oxalicum* HP7-1 genomic DNA, were introduced into the plasmid pGADT7, respectively, to generate the pGADT7-*cxrA*, pGADT7-*cxrA*_Δ1-60_^R94A^, pGADT7-*prmt2* and pGADT7-*prmt3*. Subsequently, these relevant recombinant plasmids were co-transformed into Y2HGold competent cells. The SD/-Trp/-Leu/-His/-Ade medium with 100 ng/mL AbA and 1 M X-α-Gal (chromogenic substrate) was used to screen for positive transformants. Y2HGold cells containing pGBKT7-p53 and pGADT7-T was the positive control, and Y2HGold containing pGBKT7-Lam and pGADT7-T was the negative control.

### GST-pulldown assay

*In vitro* protein-protein interactions were investigated using the GST-pulldown assay, as described previously [5]. Western blotting was employed to test the target protein with anti-GST and anti-His antibodies (TransGen Biotech Co., Ltd.).

### Construction of recombinant *P*. *oxalicum* strains

All mutants were constructed based on homologous recombination techniques, as described previously [2]. The primers used for mutant construction are shown in S1 Table.

### Colony phenotype observation

*P*. *oxalicum* colonies on agar plates containing different carbon sources were photographed with a digital camera (EOS 6D; Canon Inc., Tokyo, Japan).

### RNA-Sequencing and RT-qPCR assays

RNA-sequencing was performed by Frasergen (Wuhan, China) where the sequenced data were analyzed as described previously [30]. Briefly, the software packages SOAPnuke (v 2.1.0) [31] and HISAT2 (v 2.2.1) [32] were used for quality control of data and mapping to the *P*. *oxalicum* genome, respectively [13]. Gene expression was analyzed with both RSEM (v1.3.3) [33] and Bowtie2 (v2.3.5) [34], then visualized via the number of fragments per kilobase of exon per million mapped reads. Differentially expressed genes were searched with the DESeq2 tool [35], using false discovery rate (FDR) < 0.05 and |Log2 fold change| > 1.0 as thresholds.

The RT-qPCR assay was performed with the ChamQ Universal SYBR qPCR Master Mix, following the manufacturer’s instructions (Vazyme Biotech, Nanjing, China). The internal reference gene was *POX_d06005* (encoding actin), generated by PCR amplification (S1 Table). The relative expression of the tested gene in *P*. *oxalicum* mutants was normalized to that of the background strain Δ*ku70*;*hph*^R+^, calculated by the 2^−ΔΔCT^ method [36]. All experiments were replicated at least three times.

### Enzyme activity assays

The cellulase and xylanase activities of *P*. *oxalicum* strains were measured as described previously [2]. Filter paper cellulase and carboxymethyl cellulase were assayed with Whatman No. 1 filter paper (GE Healthcare Life Sciences, Little Chalfont, UK) and 1% carboxymethyl cellulose (Sigma-Aldrich, St. Louis, MO) as substrates, respectively. Xylanase activity was assayed with beechwood xylan (Megazyme International, Bray, Ireland) as substrate. An enzyme activity unit (U) was defined as the quantity of enzyme required to produce 1 μmoL of reducing sugar per min from the substrate. Cellulase and xylanase production by *P*. *oxalicum* was defined as units of enzyme activity per milliliter of crude culture or per gram of intracellular protein extracted from mycelia.

### Extraction of intracellular protein from *P*. *oxalicum* mycelia

The harvested mycelia separated from MMM containing Avicel inoculated with *P*. *oxalicum* were ground into powder after adding liquid nitrogen. The powder was dissolved in protein extraction buffer comprising of 0.5 mM phenylmethylsulfonyl fluoride (PMSF), 8.5 g/L NaCl, 0.2 g/L NaH_2_PO_4_, 2.2 g/L Na_2_HPO_4_ and 0.4 g/L ethylene diamine tetraacetic acid (EDTA), then the supernatant containing mycelial proteins was collected by centrifugation. Protein concentration was measured by Bradford Assay Kit (Pierce Biotechnology, Rockford, IL, USA).

### Measurement of methyltransferase production by *P*. *oxalicum*

Methyltransferase production of *P*. *oxalicum* mutant Δ*prmt3*;*G418*^R+^, background strain Δ*ku70*;*hph*^R+^ and complementation strain C*prmt3*;*ble*^R+^ was assayed using a histone methyltransferase ELISA assay kit (#YJ608911; mlBio, Shanghai, China), following the manufacturer’s instructions.

### *In vitro* methylation experiment

*In vitro* methylation assay was performed as described previously [37]. In brief, the GST-tagged CxrA and His-tagged methyltransferase were expressed in *E*. *coli* and purified. The purified GST-CxrA and methyltransferase were mixed with S-adenosyl-L-methionine as methyl donor, and reacted for 2 h at 30°C. Western blotting was used to detect substrate methylation with the corresponding antibodies including anti-mono methyl arginine, anti-symmetric di-methyl arginine, and anti-asymmetric di-methyl arginine.

### Protein sequence analysis

Protein sequences from *P*. *oxalicum* and the other related species were analyzed on and downloaded from, the NCBI website (https://www.ncbi.nlm.nih.gov/). Conserved domains were identified using the SMART database (http://smart.embl.de/). The cladogram of relative proteins was built using the Neighbor-joining method and the Poisson correction model in MEGA version X [38]. Alignment of protein sequences was performed with the multiple Sequence Alignment tool, ClustalW, on MUSCLE (https://www.ebi.ac.uk/Tools/msa/muscle/).

### Investigation of protein subcellular localization

The subcellular localization of target proteins in the mycelia of *P*. *oxalicum* cultured in Avicel medium was determined with green fluorescent protein (GFP) as the reporter, as described previously [5].

### LC-MS/MS analysis

The LC-MS/MS assay to detect post-translational modification of CxrA was performed as described previously [5]. In brief, the fusion protein CxrA-GFP was precipitated with anti-GFP antibody (TransGen Biotech Co., Ltd.) and purified by BeaverBeads Proute from an A (or A/G) Immunoprecipitation Kit (Beaver Biomedical Engineering Co., Ltd. Suzhou, China). The isolated PoxCxrA-GFP was analyzed on a liquid chromatography system (Waters, Milford, MA, USA) coupled with a Thermo Scientific LTQ-Orbitrap Mass Spectrometer (Thermo Fisher Scientific, Bremen, Germany).

### Data statistical analysis

The obtained data in this study was statistically analyzed using Microsoft Excel (Office 2019) (Microsoft, Redmond, WA, USA) and SPSS (IBM, Armonk, NY, USA) with Student’s *t*-test and one-way ANOVA, respectively.

## Supporting information

S1 Fig **Construction strategy for *P. oxalicum* mutants involving introduction of truncated *cxrA* genes into Δ*cxrA;G418^R+^* (A) and confirmation by PCR analysis (B-E).** (**B**) PCR production of *POX_d05452* with primers POX_d05452-F/POX_d05452-R. (**C**) DNA fragment with primers POX_d05452-LF/Ble-VR. (**D**) DNA fragment with primers Ble-VF/POX_d05452-LR. M: 1 kb DNA marker; 1: ddH_2_O; 2: Δ*ku70;hph*^R+^, 3: *CcxrA;ble*^R+^, 4: 1–60; 5: 1–206; 6:1–591; 7: 17–733; 8: 61–733; 9: 61–206. (**E**) PCR verification of mutant R94A;*ble*^R+^. M: 1 kb DNA marker; 1: ddH_2_O; 2: Δ*ku70;hph*^R+^, 3: *R94A;ble*^R+^. Left panel indicates amplification of DNA fragment with primers POX_d05452-F/POX_d05452-R; Middle panel shows PCR products with primers POX_d05452-LF/Ble-VR; Right panel shows PCR amplification of DNA fragment with primers Ble-VF/POX_d05452-LR. The bottom panel shows verification of DNA sequence. M: 1 kb DNA marker; 1–12: Transformants; +: Δ*ku70;hph*^R+^;–: ddH_2_O.
(TIF)

S2 Fig Phenotypes of *P*. *oxalicum* strains on solid plates containing various carbon sources. These fungal strains were cultured for 4 d. PDA: Potato dextrose agar; CMC: Carboxymethyl cellulose.
(TIF)

S3 Fig Construction of *P*. *oxalicum* overexpression strain O*cxrA-his*;*G418*^R+^ and the production of cellulase and xylanase by the constructed strains. (**A**) Schematic illustration showing construction strategy. (**B**) PCR confirmation. Upper panel shows PCR production of *cxrA*; Middle panel indicates DNA fragments with primers POX_d05452-F/G418-VR; Bottom panel presents DNA fragments with primers POX_d05452-F/G418-VR. (**C**) Filter paper cellulase (FPase) production. (**D**) Carboxymethyl cellulase (CMCase) production. (**E**) xylanase production. Fungal strains were pre-grown in glucose medium for 24 h, then transferred into Avicel medium for 2–4 d. Enzyme production is normalized to the intracellular proteins of mycelia representing fungal growth. (**F**) Relative expression of *cxrA* in both O*cxrA-his*;*G418*^R+^ and Δ*ku70*;*hph*^R+^. Total RNA as template was extracted from fungal mycelia harvested after culture on Avicel for 48 h. ** *p* < 0.01 and * *p* < 0.05 indicate significant differences between the overexpression strain and background strain, assessed by Student’s *t*-test.
(TIF)

S4 Fig LC-MS/MS assay indicating post-translational modification of CxrA in the presence of Avicel. (**A**) Amino acid sequence; residues in yellow were modified. Red P, M and A represent phosphorylation, acetylation, and methylation. (**B**) Oligopeptides with red color were identified by LC-MS/MS.
(PDF)

S5 Fig Autoactivation experiment on the full-length CxrA in *Saccharomyces cerevisiae*. Serial dilutions of yeast Y2HGold cells carrying pGBKT7-*cxrA* and pGBKT7, pGBKT7+pGADT7-p53, pGBKT7+pGADT7-Lam as controls were cultured on SD/-Trp, SD/-Trp/X-α-Gal and SD/-Trp/X-α-Gal/AbA at 30°C for 4 d.
(TIF)

S6 Fig **Autoactivation detection of the bait CxrA_Δ1–60_ (A) and determination of CxrA_Δ1–60_ toxicity to yeast cells (B).** Serial dilutions of yeast Y2HGold cells carrying pGBKT7-*cxrA*_Δ1–60_ and pGBKT7 as control were cultured on SD/-Trp, SD/-Trp/X-α-Gal and SD/-Trp/X-α-Gal/AbA at 30°C for 4 d.
(TIF)

S7 Fig **Construction strategy (A–F) and PCR verification (G–Y) of *P. oxalicum* mutants used in this study.** These strains include the overexpression strain *OPOX_f08428;G418*^R+^ (**A; G–J**), mutants Δ*POX_b03080;G418*^R+^ (**B; K–M**), Δ*POX_d05270;G418*^R+^ (**B; N–P**), Δ*POX_e06662;G418*^R+^ (**B; Q–S**), complementation strain *Cprmt3;ble*^R+^ (**C; T–V**), *Oprmt3-gfp;G418*^R+^ (**D; W**), *OcxrA-gfp;G418*^R+^ (**E; X**) and Δ*prmt3;cxrA-gfp;G418*^R+^;*ble*^R+^ (**F; Y**). (**G; T**) PCR amplification of *POX_d05452* with primer pair POX_d05452-F/POX_d05452-R. (**H**), (**L**), (**O**) and (**R**) DNA fragments with primers Target-LF/G418-VR. (**I**) PCR production of G418 resistance gene. (**J**), (**M**), (**P**) and (**S**) DNA fragments with primers G418-VF/Target-RR. (**K**), (**N**) and (**Q**) PCR amplification of target genes. M: 1 kb DNA marker; 1–3: Three transformants; 4: Δ*ku70;hph*^R+^; 5: ddH2O. (**T**) PCR amplification of POX_d05452. (**U**) PCR products with primers POX_d05452-LF/Ble-VR. (**V**) PCR amplification of DNA fragment with primers Ble-VF/POX_d05452-LR. M: 1 kb DNA marker; 1: ddH2O; 2: Δ*ku70;hph*^R+^; 3: *Cprmt3;ble*^R+^. In panel W, X and Y, upper panel shows amplification of DNA fragment with primers POX_d05452-F/POX_d05452-R; Middle panel shows PCR products with primers POX_d05452-LF/Ble-VR; Bottom panel shows PCR amplification of DNA fragment with primers Ble-VF/POX_d05452-LR. M: 1 kb DNA marker; 1: ddH_2_O; 2: Δ*ku70*; 3: *Oprmt3-gfp;G418*^R+^, *OcxrA-gfp;G418*^R+^ or Δ*prmt3;cxrA-gfp;G418*^R+^;*ble*^R+^. *pepA* (*POX_d05452*): aspartic protease gene; G418:; *ble*: bleomycin antibiotics gene; *pepA*-L: left-flanking sequence of gene *pepA*; *pepA*-R: right-flanking sequence of gene *pepA*; *pepA*-P: the promoter region of gene *pepA*; *pepA*-T: the terminus region of gene *pepA*; ORF: open reading frame; *POX_f08428*-P: the promoter region of gene *POX_f08428*; *POX_f08428*-T: the terminus region of gene *POX_f08428*; *cxrA*-P: the promoter region of gene *cxrA*; *cxrA*-T: the terminus region of gene *cxrA*.
(TIF)

S8 Fig Filter paper cellulase production of *P*. *oxalicum* mutants Δ*POX_d05270*;*G418*^R+^ and Δ*POX_e06662*;*G418*^R+^. Fungal strains were cultivated on Avicel for 2–4 days after transfer from glucose. Data values indicate means ± standard deviation.
(TIF)

S9 Fig Relative expression of the genes *POX_f08428*, *prmt3 and cxrA* in *P*. *oxalicum* overexpression strains O*POX_f08428*;*G418*^R+^, O*prmt3-gfp*;*G418*^R+^ and O*cxrA-gfp*;*G418*^R+^ as compared with background strain Δ*ku70*, respectively. *P*. *oxalicum* strains pre-grow in glucose medium for 24 h, and the harvested mycelia are transferred into Avicel medium and cultured for 48 h. Gene expression in the overexpression strain is normalized to the level of Δ*ku70*;*hph*^R+^. Data points show mean ± standard deviation. ** *p* < 0.01 and * *p* < 0.05 indicate significant differences between the overexpression strain and background strain, assessed by Student’s *t*-test.
(TIF)

S10 Fig Autoactivation experiment of the PRMT2 in *Saccharomyces cerevisiae*. Serial dilutions of yeast Y2HGold cells carrying pGBKT7-*prmt2* and pGBKT7, pGBKT7+pGADT7-p53, pGBKT7+pGADT7-Lam as controls were cultured on SD/-Trp, SD/-Trp/X-α-Gal and SD/-Trp/X-α-Gal/AbA at 30°C for 4 d.
(TIF)

S11 Fig *In vitro* methylation assay of CxrA_Δ1–60_ by PRMT3. anti-MMA: mono methyl arginine antibody; anti-ADMA: asymmetric dimethyl arginine antibody; anti-DMA: dimethyl arginine antibody; SAM: S-adenosyl-methionine. “+” and “−” indicate the presence or absence of the test protein.
(TIF)

S12 Fig Pearson’s correlation heatmap of Δ*prmt3*;*G418*^R+^ and Δ*cxrA*;*G418*^R+^ transcriptomes compared with that of the background strain Δ*ku70*;*hph*^R+^, grown on Avicel. Total RNA was extracted from the mycelia of each strain after culture in Avicel medium for 24 h, following a transfer from glucose.
(TIF)

S1 Table Primers used in this study. (DOCX)

S1 Dataset List of 997 differentially expressed genes in Δ*prmt3*;*G418*^R+^ compared with the background strain Δ*ku70*;*hph*^R+^, grown on Avicel. (XLSX)

S2 Dataset List of 2552 differentially expressed genes in Δ*cxrA*;*G418*^R+^ compared with the background strain Δ*ku70*;*hph*^R+^, grown on Avicel. (XLSX)

S3 Dataset Data from Figs 1–5, S3, S8 and S9. (XLSX)
