# Supplementary figures and images for: Arginine methyltransferases PRMT2 and PRMT3 are essential for biosynthesis of plant-polysaccharide-degrading enzymes in Penicillium oxalicum
Source: PLoS Genet. 2023 Jul 31;19(7):e1010867. doi: 10.1371/journal.pgen.1010867 (PMC10414604; doi:10.1371/journal.pgen.1010867)

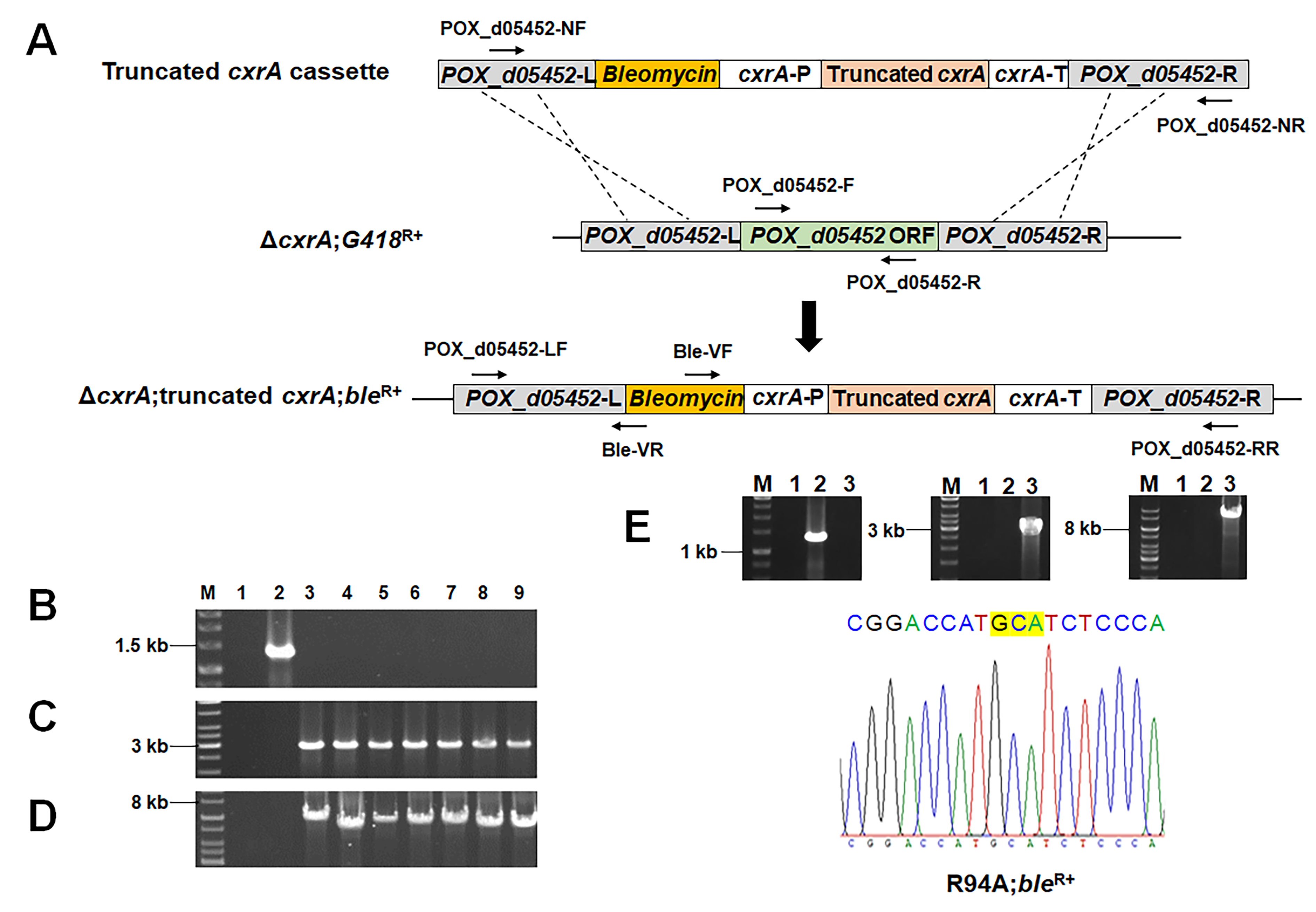

Supplement: S1 Fig — Construction strategy for P. oxalicum mutants involving introduction of truncated cxrA genes into ΔcxrA;G418R+ (A) and confirmation by PCR analysis (B-E). (B) PCR production of POX_d05452 with primers POX_d05452-F/POX_d05452-R. (C) DNA fragment with primers POX_d05452-LF/Ble-VR. (D) DNA fragment with primers Ble-VF/POX_d05452-LR. M: 1 kb DNA marker; 1: ddH2O; 2: Δku70;hphR+, 3: CcxrA;bleR+, 4: 1–60; 5: 1–206; 6:1–591; 7: 17–733; 8: 61–733; 9: 61–206. (E) PCR verification of mutant R94A;bleR+. M: 1 kb DNA marker; 1: ddH2O; 2: Δku70;hphR+, 3: R94A;bleR+. Left panel indicates amplification of DNA fragment with primers POX_d05452-F/POX_d05452-R; Middle panel shows PCR products with primers POX_d05452-LF/Ble-VR; Right panel shows PCR amplification of DNA fragment with primers Ble-VF/POX_d05452-LR. The bottom panel shows verification of DNA sequence. M: 1 kb DNA marker; 1–12: Transformants; +: Δku70;hphR+;–: ddH2O. (TIF) [file pgen.1010867.s001.tif]

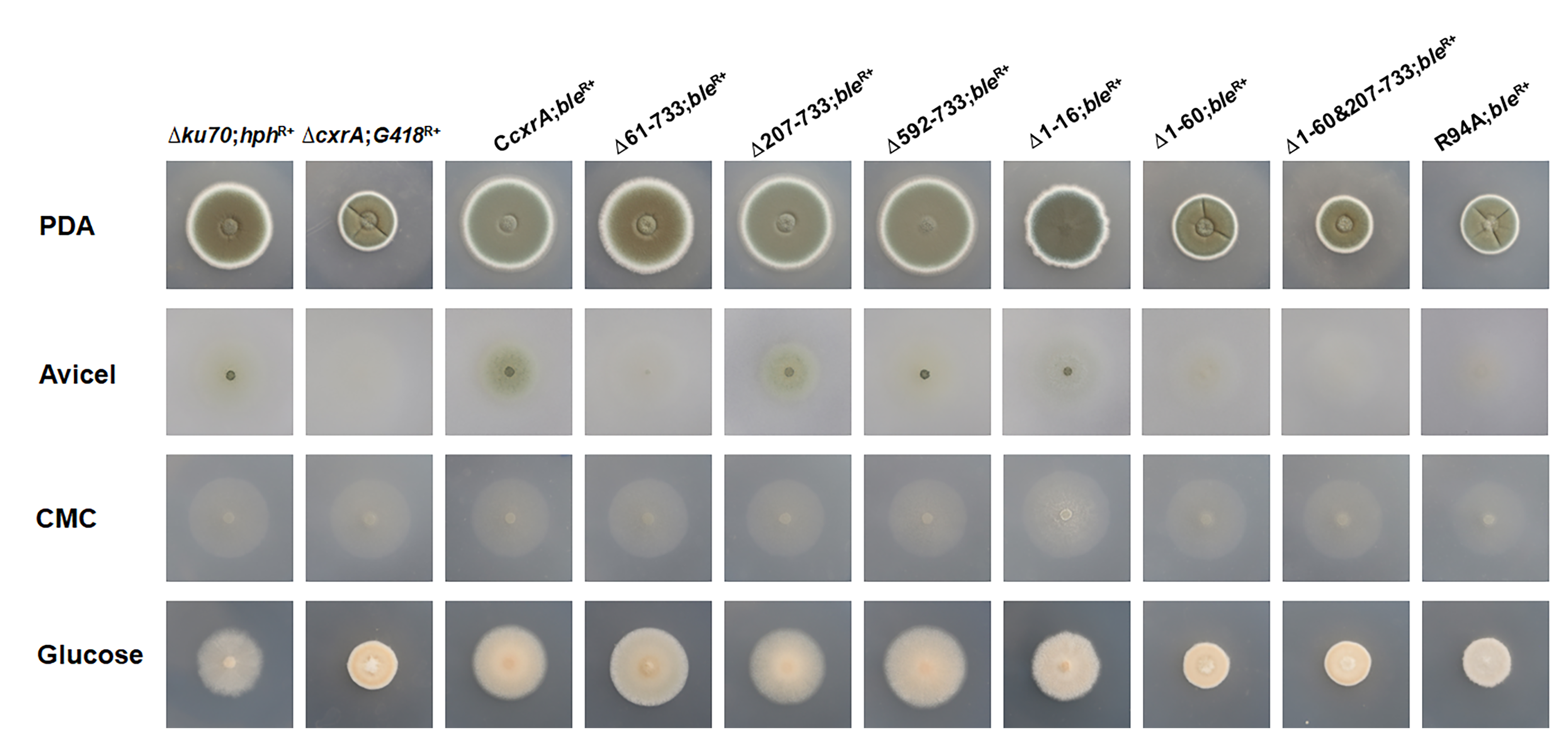

Supplement: S2 Fig — These fungal strains were cultured for 4 d. PDA: Potato dextrose agar; CMC: Carboxymethyl cellulose. (TIF) [file pgen.1010867.s002.tif]

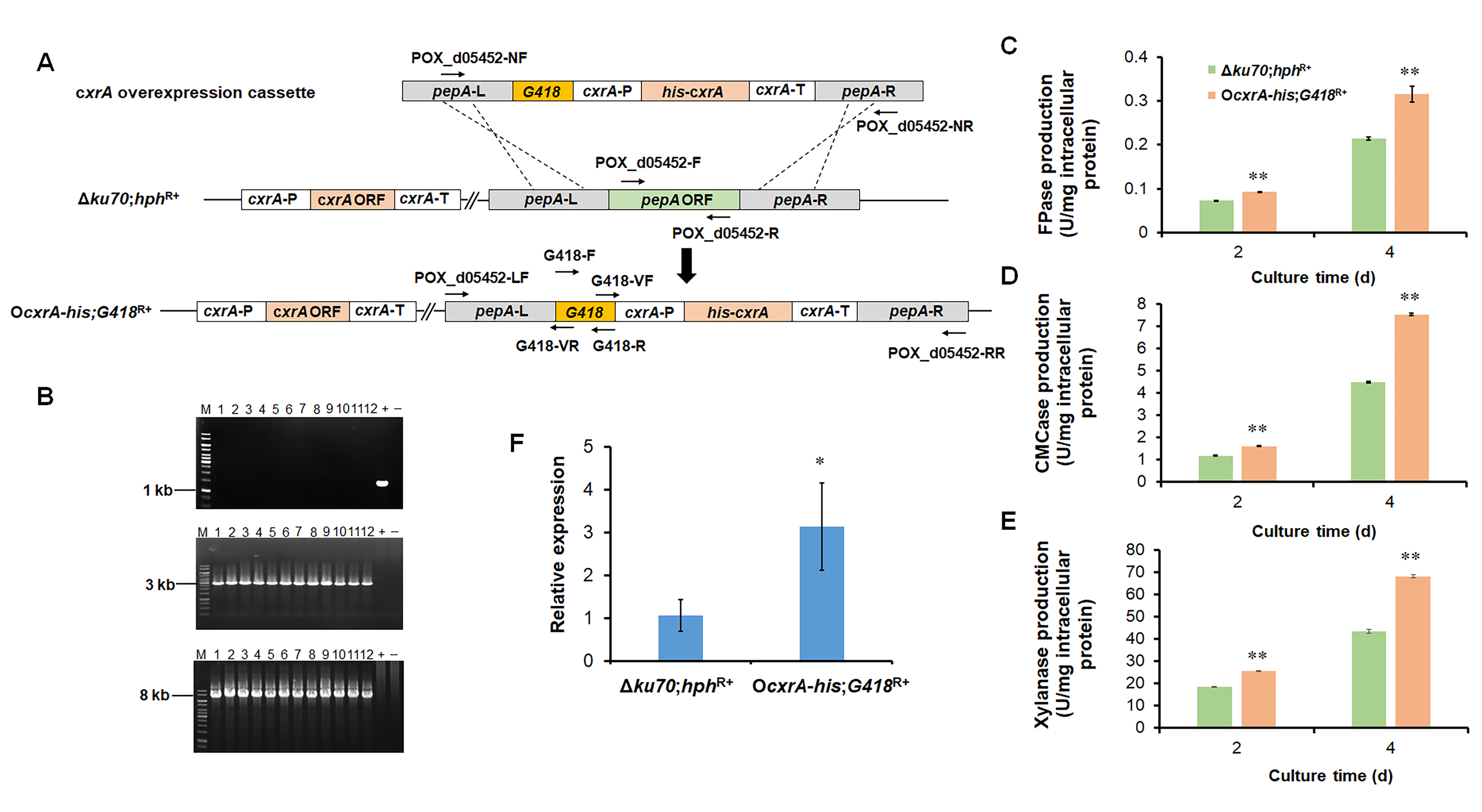

Supplement: S3 Fig — (A) Schematic illustration showing construction strategy. (B) PCR confirmation. Upper panel shows PCR production of cxrA; Middle panel indicates DNA fragments with primers POX_d05452-F/G418-VR; Bottom panel presents DNA fragments with primers POX_d05452-F/G418-VR. (C) Filter paper cellulase (FPase) production. (D) Carboxymethyl cellulase (CMCase) production. (E) xylanase production. Fungal strains were pre-grown in glucose medium for 24 h, then transferred into Avicel medium for 2–4 d. Enzyme production is normalized to the intracellular proteins of mycelia representing fungal growth. (F) Relative expression of cxrA in both OcxrA-his;G418R+ and Δku70;hphR+. Total RNA as template was extracted from fungal mycelia harvested after culture on Avicel for 48 h. ** p < 0.01 and * p < 0.05 indicate significant differences between the overexpression strain and background strain, assessed by Student’s t-test. (TIF) [file pgen.1010867.s003.tif]

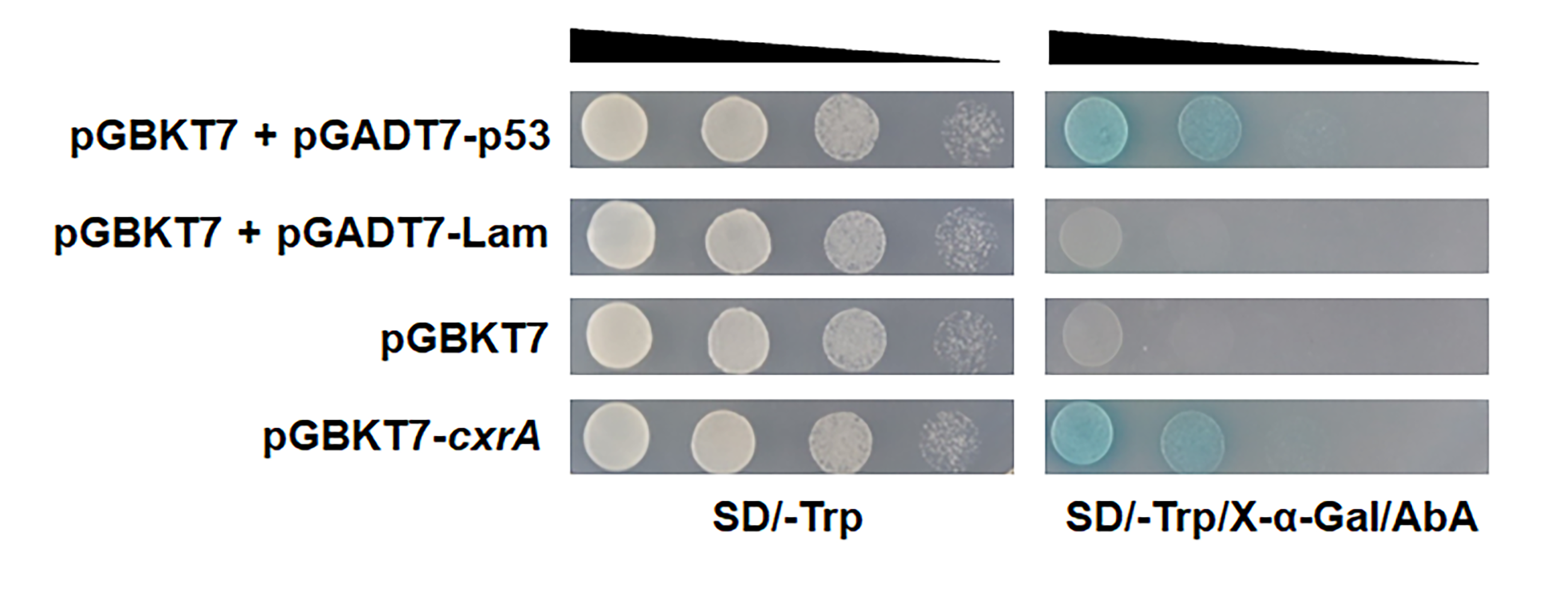

Supplement: S5 Fig — Serial dilutions of yeast Y2HGold cells carrying pGBKT7-cxrA and pGBKT7, pGBKT7+pGADT7-p53, pGBKT7+pGADT7-Lam as controls were cultured on SD/-Trp, SD/-Trp/X-α-Gal and SD/-Trp/X-α-Gal/AbA at 30°C for 4 d. (TIF) [file pgen.1010867.s005.tif]

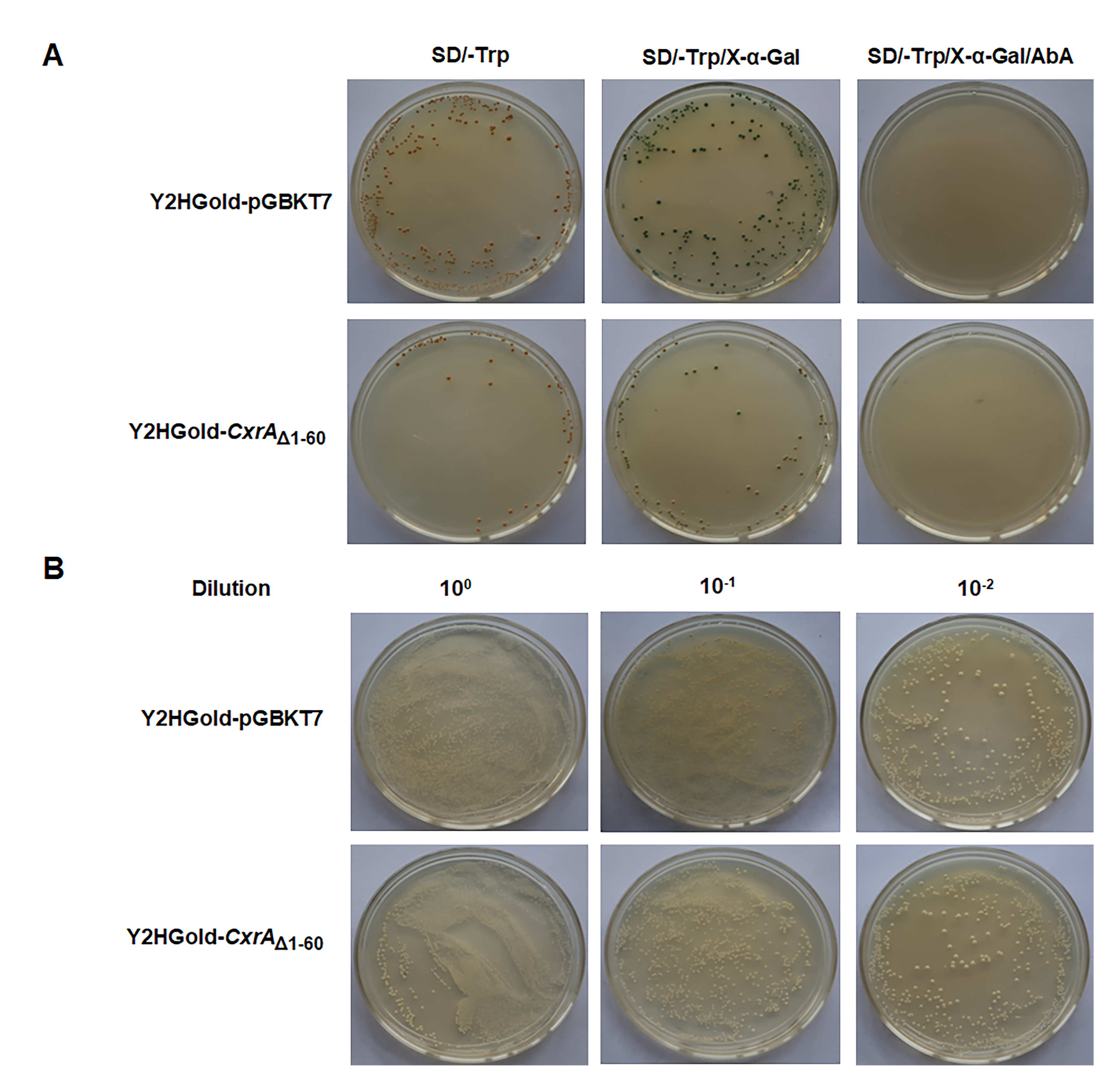

Supplement: S6 Fig — Autoactivation detection of the bait CxrAΔ1–60 (A) and determination of CxrAΔ1–60 toxicity to yeast cells (B). Serial dilutions of yeast Y2HGold cells carrying pGBKT7-cxrAΔ1–60 and pGBKT7 as control were cultured on SD/-Trp, SD/-Trp/X-α-Gal and SD/-Trp/X-α-Gal/AbA at 30°C for 4 d. (TIF) [file pgen.1010867.s006.tif]

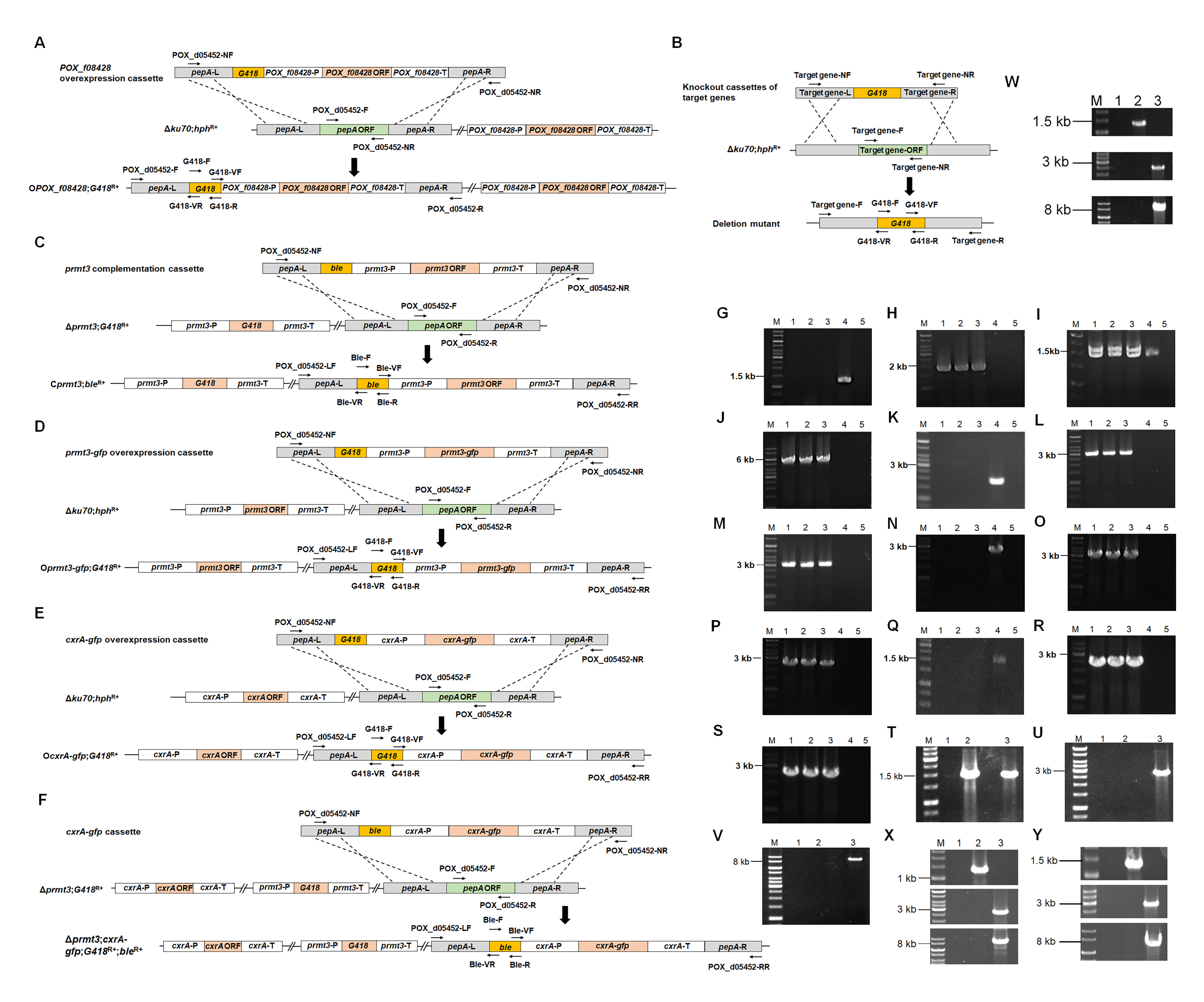

Supplement: S7 Fig — Construction strategy (A–F) and PCR verification (G–Y) of P. oxalicum mutants used in this study. These strains include the overexpression strain OPOX_f08428;G418R+ (A; G–J), mutants ΔPOX_b03080;G418R+ (B; K–M), ΔPOX_d05270;G418R+ (B; N–P), ΔPOX_e06662;G418R+ (B; Q–S), complementation strain Cprmt3;bleR+ (C; T–V), Oprmt3-gfp;G418R+ (D; W), OcxrA-gfp;G418R+ (E; X) and Δprmt3;cxrA-gfp;G418R+;bleR+ (F; Y). (G; T) PCR amplification of POX_d05452 with primer pair POX_d05452-F/POX_d05452-R. (H), (L), (O) and (R) DNA fragments with primers Target-LF/G418-VR. (I) PCR production of G418 resistance gene. (J), (M), (P) and (S) DNA fragments with primers G418-VF/Target-RR. (K), (N) and (Q) PCR amplification of target genes. M: 1 kb DNA marker; 1–3: Three transformants; 4: Δku70;hphR+; 5: ddH2O. (T) PCR amplification of POX_d05452. (U) PCR products with primers POX_d05452-LF/Ble-VR. (V) PCR amplification of DNA fragment with primers Ble-VF/POX_d05452-LR. M: 1 kb DNA marker; 1: ddH2O; 2: Δku70;hphR+; 3: Cprmt3;bleR+. In panel W, X and Y, upper panel shows amplification of DNA fragment with primers POX_d05452-F/POX_d05452-R; Middle panel shows PCR products with primers POX_d05452-LF/Ble-VR; Bottom panel shows PCR amplification of DNA fragment with primers Ble-VF/POX_d05452-LR. M: 1 kb DNA marker; 1: ddH2O; 2: Δku70; 3: Oprmt3-gfp;G418R+, OcxrA-gfp;G418R+ or Δprmt3;cxrA-gfp;G418R+;bleR+. pepA (POX_d05452): aspartic protease gene; G418:; ble: bleomycin antibiotics gene; pepA-L: left-flanking sequence of gene pepA; pepA-R: right-flanking sequence of gene pepA; pepA-P: the promoter region of gene pepA; pepA-T: the terminus region of gene pepA; ORF: open reading frame; POX_f08428-P: the promoter region of gene POX_f08428; POX_f08428-T: the terminus region of gene POX_f08428; cxrA-P: the promoter region of gene cxrA; cxrA-T: the terminus region of gene cxrA. (TIF) [file pgen.1010867.s007.tif]

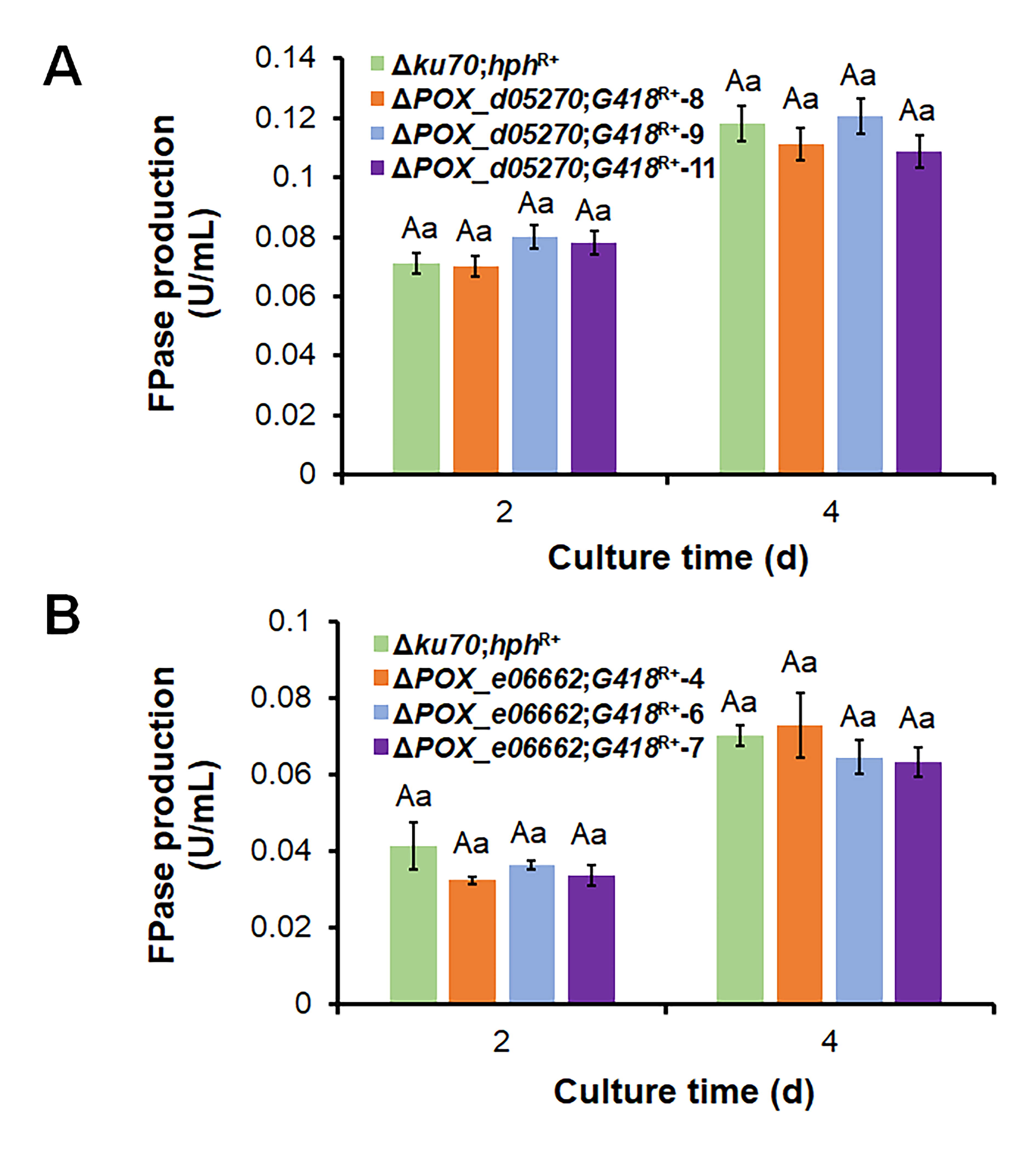

Supplement: S8 Fig — Fungal strains were cultivated on Avicel for 2–4 days after transfer from glucose. Data values indicate means ± standard deviation. (TIF) [file pgen.1010867.s008.tif]

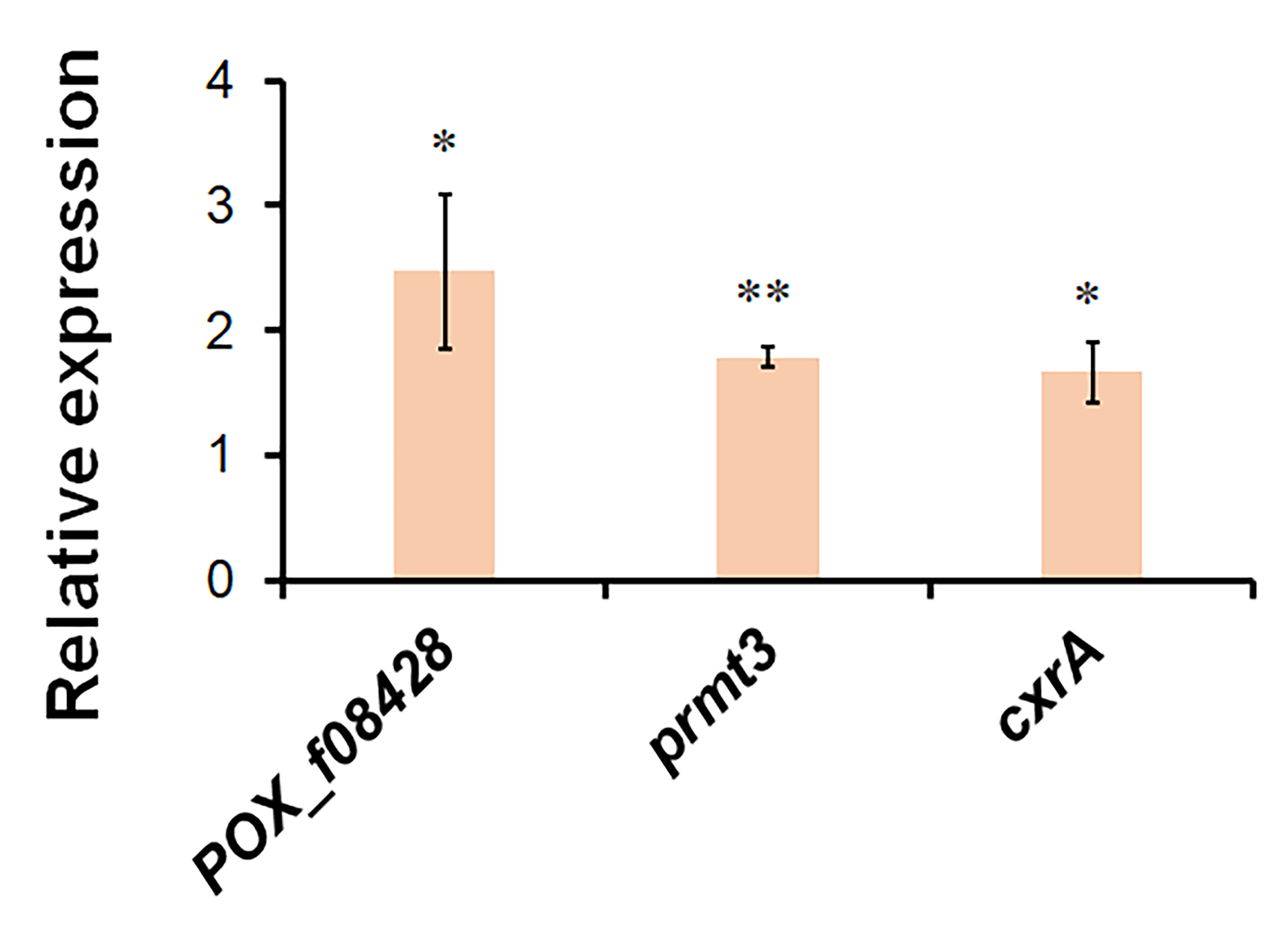

Supplement: S9 Fig — P. oxalicum strains pre-grow in glucose medium for 24 h, and the harvested mycelia are transferred into Avicel medium and cultured for 48 h. Gene expression in the overexpression strain is normalized to the level of Δku70;hphR+. Data points show mean ± standard deviation. ** p < 0.01 and * p < 0.05 indicate significant differences between the overexpression strain and background strain, assessed by Student’s t-test. (TIF) [file pgen.1010867.s009.tif]

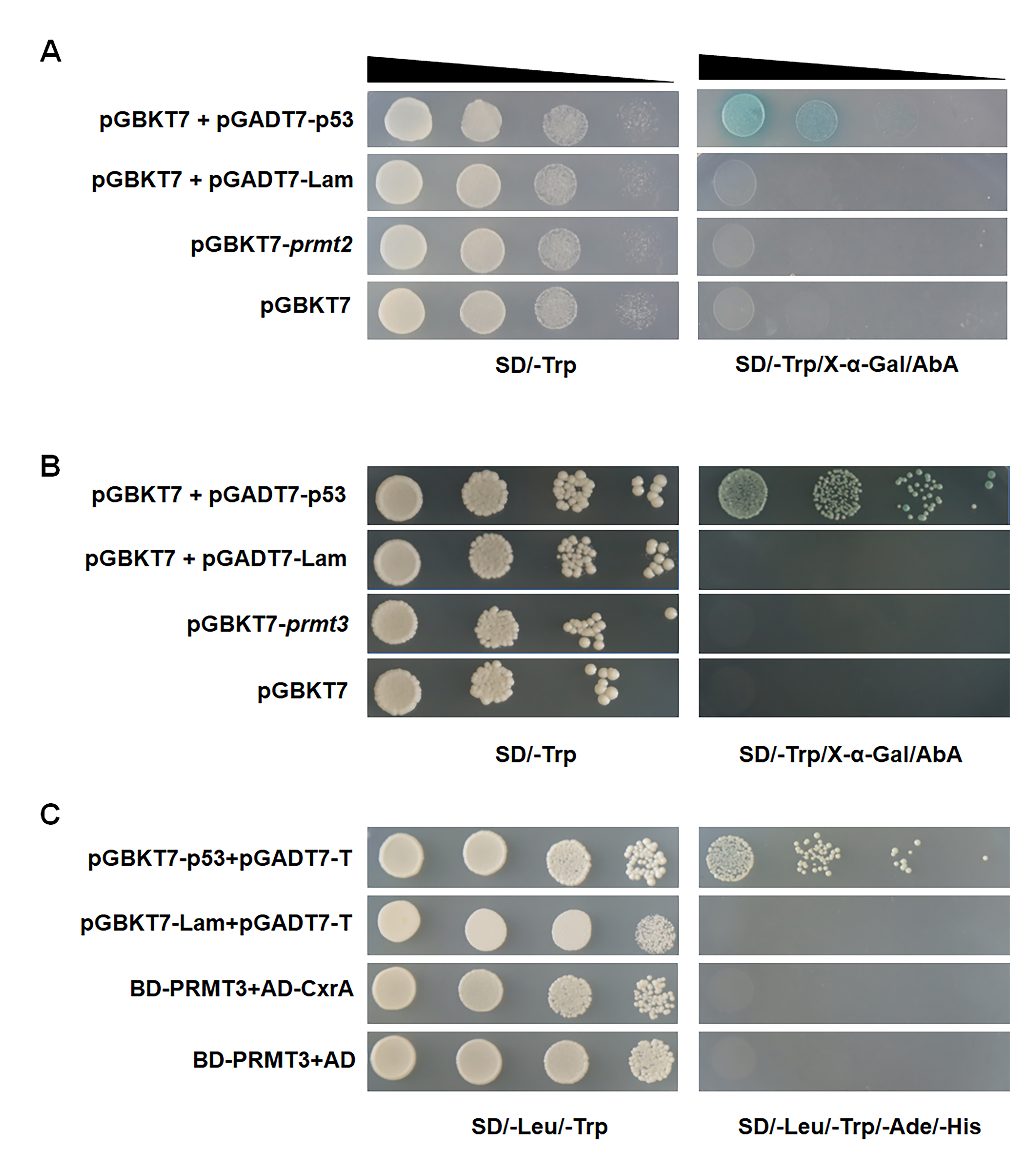

Supplement: S10 Fig — Serial dilutions of yeast Y2HGold cells carrying pGBKT7-prmt2 and pGBKT7, pGBKT7+pGADT7-p53, pGBKT7+pGADT7-Lam as controls were cultured on SD/-Trp, SD/-Trp/X-α-Gal and SD/-Trp/X-α-Gal/AbA at 30°C for 4 d. (TIF) [file pgen.1010867.s010.tif]

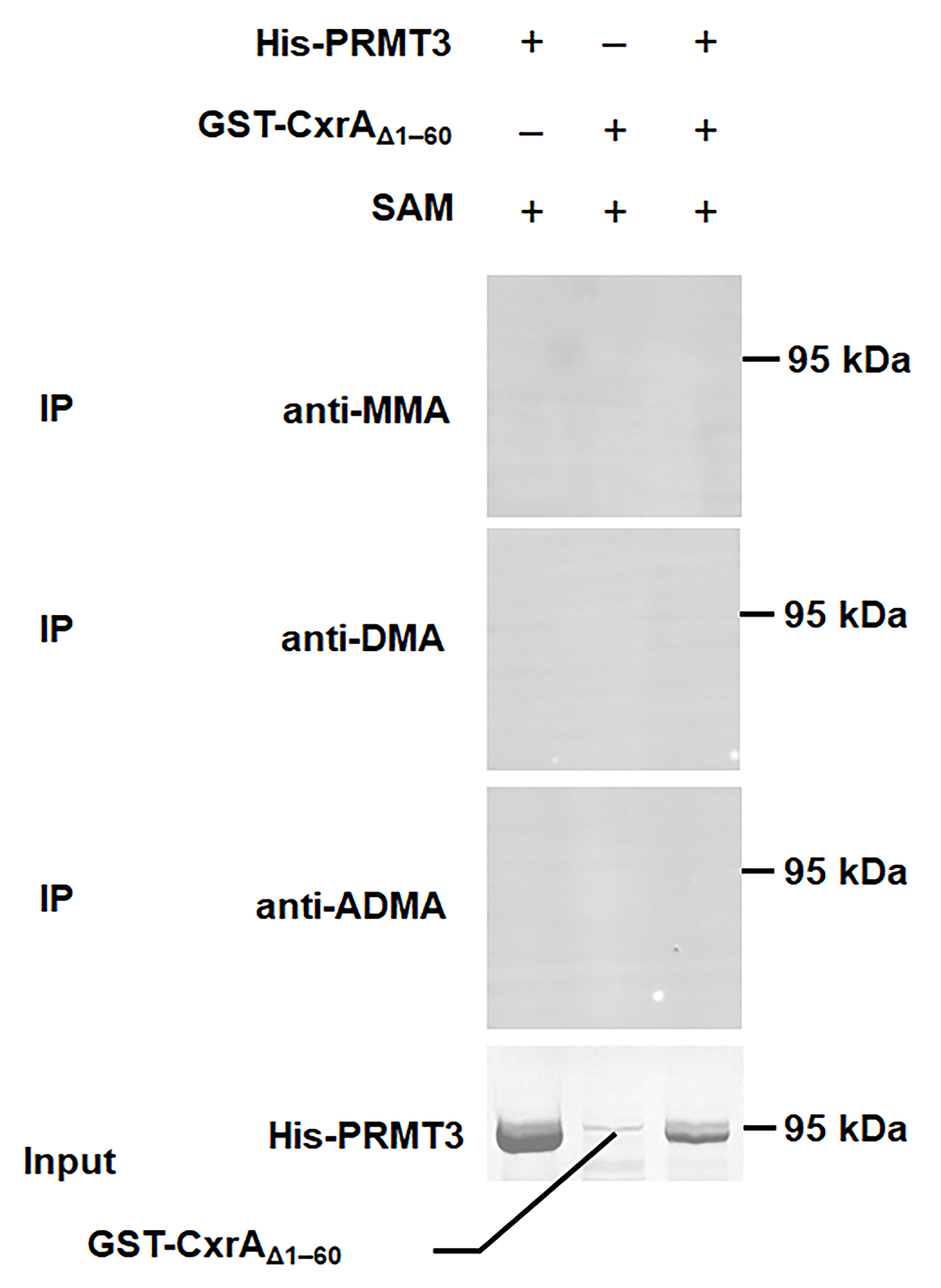

Supplement: S11 Fig — anti-MMA: mono methyl arginine antibody; anti-ADMA: asymmetric dimethyl arginine antibody; anti-DMA: dimethyl arginine antibody; SAM: S-adenosyl-methionine. “+” and “−” indicate the presence or absence of the test protein. (TIF) [file pgen.1010867.s011.tif]

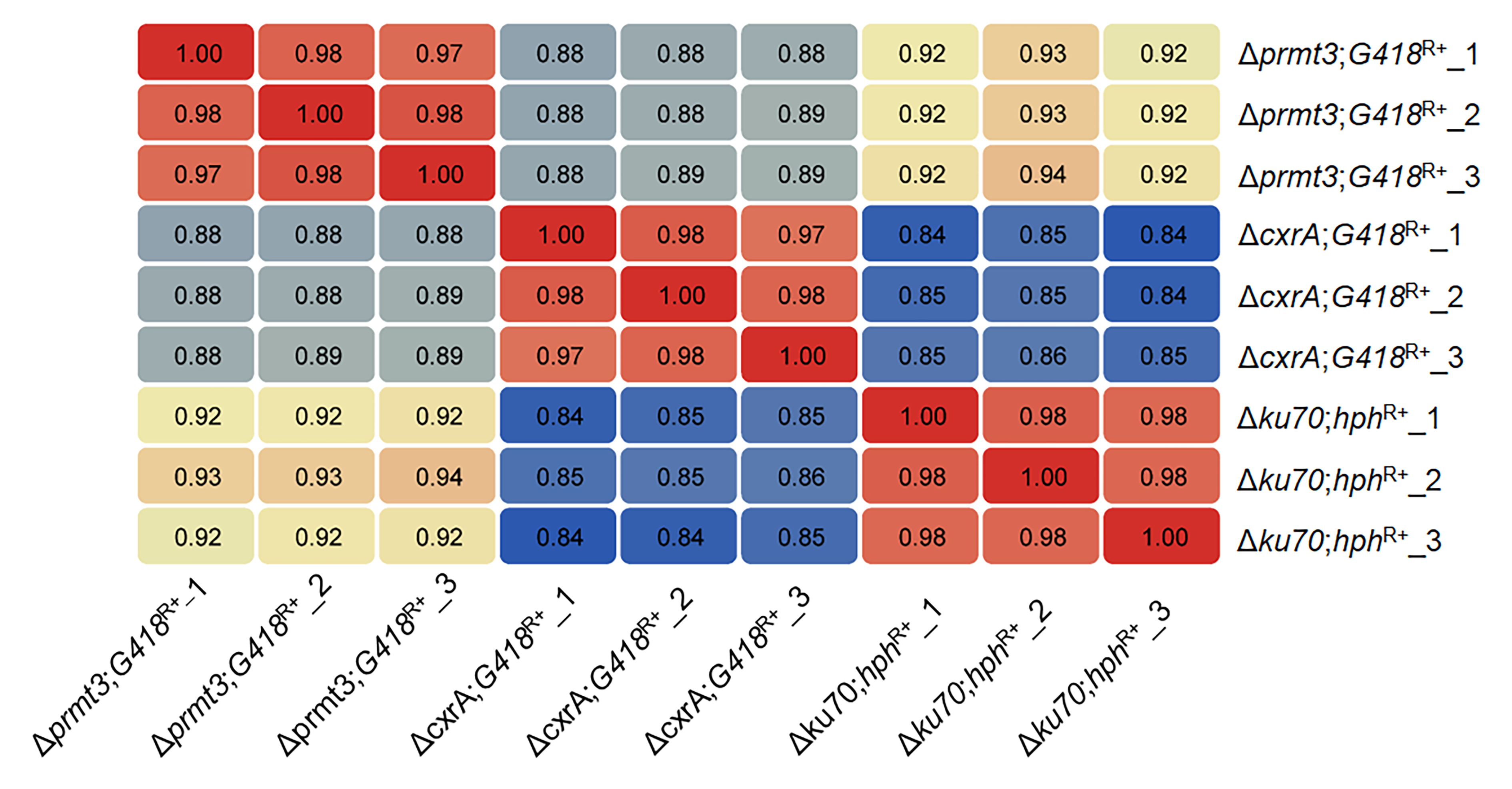

Supplement: S12 Fig — Total RNA was extracted from the mycelia of each strain after culture in Avicel medium for 24 h, following a transfer from glucose. (TIF) [file pgen.1010867.s012.tif]
