# Supplementary material for: Arginine methyltransferases PRMT2 and PRMT3 are essential for biosynthesis of plant-polysaccharide-degrading enzymes in Penicillium oxalicum
Source: PLoS Genet. 2023 Jul 31;19(7):e1010867. doi: 10.1371/journal.pgen.1010867 (PMC10414604; doi:10.1371/journal.pgen.1010867)
Supplement: S4 Fig — (A) Amino acid sequence; residues in yellow were modified. Red P, M and A represent phosphorylation, acetylation, and methylation. (B) Oligopeptides with red color were identified by LC-MS/MS. (PDF) [file pgen.1010867.s004.pdf]

A

>CxrA

A

MTTPISTSDPTATTVKPRRVLACVLCQQRKSKCDRKFPCANCVR

ANVQCEQAVRQRRRRFPERELLARLRLYESVLRQHNIKFDPLHT

M

PTADHRSPSDDGRDDLPDGAESEGTLGDTNALPSKEKPAVQPK

ALNLWHAMSQKAVNSGDDGNAGEDDENDTGFLHDNDDLRHAV

IKKAWNHMFQGQGNDHLLFGPPVGTIDLSASHPSQAHIFRLWQI

YLDNVNPLLKVTHTPTLQSRIIDAVSDIANISPTLEALMFSIYCVSL

MSLSGEQCRAMFGAARKELLASYQFACQQALRRCGVLRSSDR

ECLTALYLYLVSIRSETDPASLSSLLSIAIRIAQRMGIHVESMYSKC

SVLEAEMRRRLWWSLIIFDNRICEMSDYKTASLTPTWDCKVPLN

VNDFELQPEMKIPPVVNNRPTEMLFAIVRSELADFVRHSAFHLNF

P P M P P

TNSYLNTIAPRPPTTDLTDEAERLVSLERTLEEKYLAFCNLENPLH

FMTIWTMRGSLAKNRLLQLYSQSSNTPTPPTDAQRNAGIAHALR

MLECDTKLMTAPLARPYLWLLHFHFPFPAYIHLLQDLKKRPVEDH

ADRAWEVMSDNYEVRIMDVKQDDRPFYVVFSQIVFQAWEAREK

VARQLGTPFVLPRMVVDIRKKLTHMTTSFGQEADAAQSDGDAG

VEINADDLAMPMSIDFNVLGMAYGAGGQGPTSSGPWGYPDLPG

PGSVNVEDTNQFLVNTMEWNRLHAHGR

B

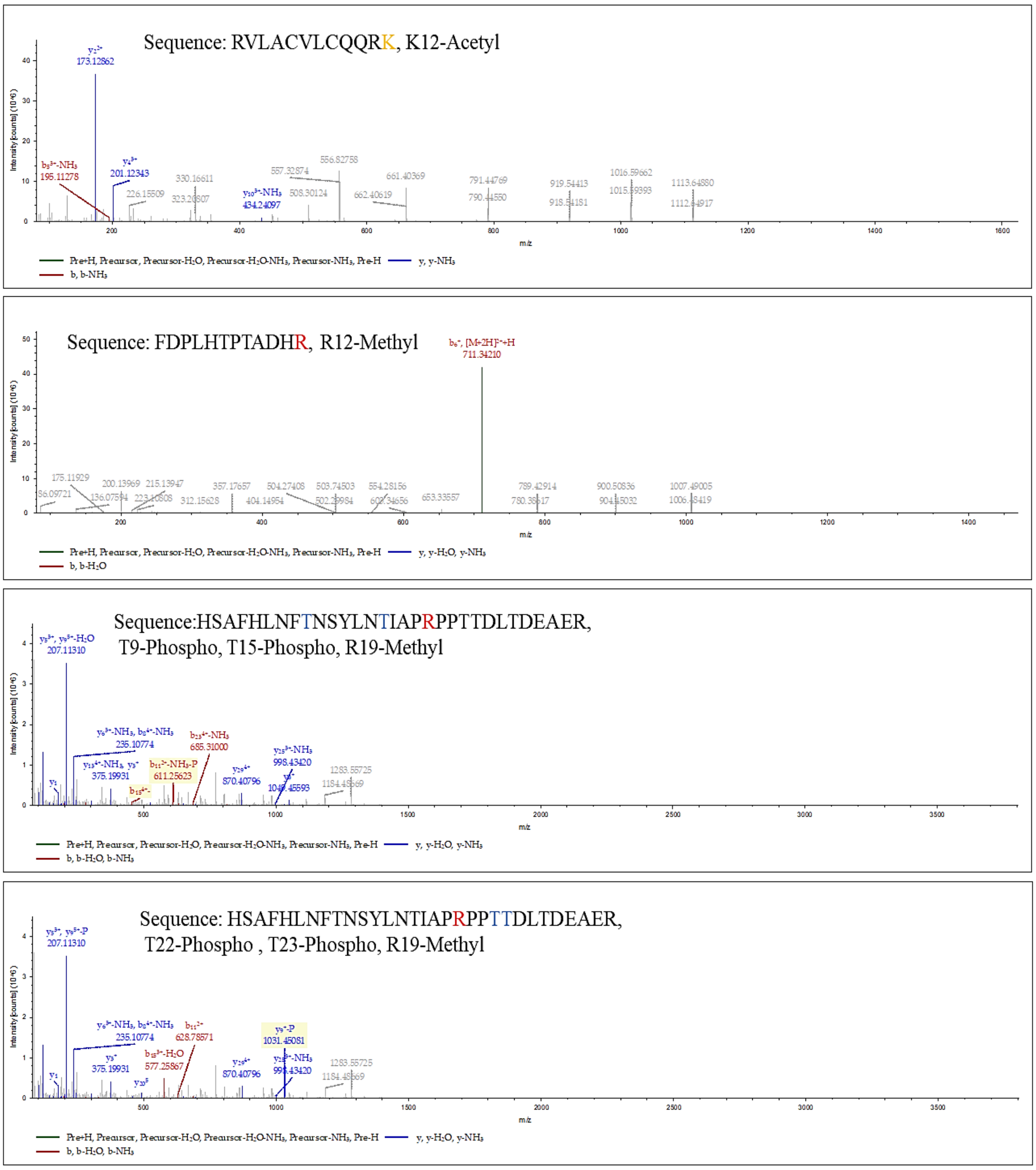

Sequence: RVLACVLCQQRK, K12-Acetyl
Intensity [counts] (10^6)
m/z
Pre+H, Precursor, Precursor-H2O, Precursor-H2O-NH3, Precursor-NH3, Pre-H
y, y-NH3
b, b-NH3
Sequence: FDPLHTPTADHR, R12-Methyl
Intensity [counts] (10^6)
m/z
Pre+H, Precursor, Precursor-H2O, Precursor-H2O-NH3, Precursor-NH3, Pre-H
y, y-H2O, y-NH3
b, b-H2O
Sequence:HSAFHLNFTNSYLNTIAPRPPTTDLTDEAER,
T9-Phospho, T15-Phospho, R19-Methyl
Intensity [counts] (10^6)
m/z
Pre+H, Precursor, Precursor-H2O, Precursor-H2O-NH3, Precursor-NH3, Pre-H
y, y-H2O, y-NH3
b, b-H2O, b-NH3
Sequence: HSAFHLNFTNSYLNTIAPRPPTTDLTDEAER,
T22-Phospho , T23-Phospho, R19-Methyl
Intensity [counts] (10^6)
m/z
Pre+H, Precursor, Precursor-H2O, Precursor-H2O-NH3, Precursor-NH3, Pre-H
y, y-H2O, y-NH3
b, b-H2O, b-NH3
