# Supplementary material for: Arginine methyltransferases PRMT2 and PRMT3 are essential for biosynthesis of plant-polysaccharide-degrading enzymes in Penicillium oxalicum
Source: PLoS Genet. 2023 Jul 31;19(7):e1010867. doi: 10.1371/journal.pgen.1010867 (PMC10414604; doi:10.1371/journal.pgen.1010867)
Supplement: S1 Table — (DOCX) [file pgen.1010867.s013.docx]

**S1 Table. Primers used in this study**

| **Application of primers** | **Primer name** | **Sequence (5’-3’)** | **Expected size of PCR production (bp)** |
| --- | --- | --- | --- |
| **Primers used for construction of the truncated *cxrA*** | | | |
| Truncation cassette of *cxrA* | CxrA-F | TCTCAAGTCGTCGGCCATGACCACGCCGATCTCTA | 2380 |
|  | CxrA-R | TTGACACTCCGCGGGCTGCCTCACCTGCCGTGAGCATGCAGC |  |
|  | CxrA_1-60_-F | TCTCAAGTCGTCGGCCATGACCACGCCGATCTCTA | 183 |
|  | CxrA_1-60_-R | CACTCCGCGGGCTGCCTTAAAATCTGCGCCGTCGCTG |  |
|  | CxrA_1-206_-F | TCTCAAGTCGTCGGCCATGACCACGCCGATCTCTA | 735 |
|  | CxrA_1-206_-R | CACTCCGCGGGCTGCCTTAATGTGACGCGGATAGATC |  |
|  | CxrA_1-591_-F | TCTCAAGTCGTCGGCCATGACCACGCCGATCTCTA | 1953 |
|  | CxrA_1-591_-R | CACTCCGCGGGCTGCCTTACACTTCGTAGTTGTCA |  |
|  | CxrA_17-733_-F | GGATCAATAGTTTCTCAAGTCGTCGGCCATGCGACGCGTGCTG | 2322 |
|  | CxrA_17-733_-R | TTGACACTCCGCGGGCTGCCTCACCTGCCGTGAGCATGCAGC |  |
|  | CxrA_61-733_-F | GGATCAATAGTTTCTCAAGTCGTCGGCCATGCCCGAGAGAGAAT | 2196 |
|  | CxrA_61-733_-R | TTGACACTCCGCGGGCTGCCTCACCTGCCGTGAGCATGCAGC |  |
|  | CxrA_61-206_-F | TACCGCTGTTGAGATCCAGATGCCCGAGAGAGAATTAT | 561 |
|  | CxrA_61-206_-R | CACTCCGCGGGCTGCCTTAATGTGACGCGGATAGATC |  |
|  | POX_d05452-LF | TGAGGGCAGGTGAAGTAG | 2458 |
|  | POX_d05452-LR | GGTAATCCTTCTTTCTAGAGATGGACGAGACGGTACGAT |  |
|  | Ble-F | CTGGATCTCAACAGCGGTA | 1755 |
|  | Ble-R | TCTAGAAAGAAGGATTACCTC |  |
|  | CxrA-P-F | TACCGCTGTTGAGATCCAGAAGCACCATCGCCAAGTCAC | 1253 |
|  | CxrA-P-R | GGCCGACGACTTGAGAAACT |  |
|  | CxrA-T-F | GGCAGCCCGCGGAGT | 905 |
|  | CxrA-T-R | TTGGTGATCTACATAAGCTCGTATCTGGTAGTGACGAAGCA |  |
|  | POX_d05452-RF | ATGTAGATCACCAAGTTGCACG | 2044 |
|  | POX_d05452-RR | AATCCACCGTCGCAATCGTCAA |  |
|  | POX_d05452-NF | ACGACTGGTAATCACGAATCA | 1557 |
|  | POX_d05452-NR | CGTCAAGGATATCGCCACTAA | 2028 |
| PCR confirmation | POX_d05452-F | ATGGTTGTCTTCAGCAAGGTTA | 1326 |
|  | POX_d05452-R | CTATGCCTGAGCAGCGAAAC |  |
|  | POX_d05452-LF | TGAGGGCAGGTGAAGTAG | 2458 |
|  | Ble-VR | ACTGAGGAATCCGCTCTTGG | 356 |
|  | Ble-VF | TGATAATAATGTCCTCGTTCCTGTC | 274 |
|  | POX_d05452-RR | AATCCACCGTCGCAATCGTCAA | 2044 |
| **Primers used for construction of *P. oxalicum* mutant R94A;*ble*^R+^** | | | |
| Complementation cassette of *cxrA*^R94A^ | POX_d05452-LF | TGAGGGCAGGTGAAGTAG | 2458 |
|  | POX_d05452-LR | GGTAATCCTTCTTTCTAGAGATGGACGAGACGGTACGAT |  |
|  | Ble-F | CTGGATCTCAACAGCGGTA | 1755 |
|  | Ble-R | TCTAGAAAGAAGGATTACCTC |  |
|  | CxrA-P-F | TACCGCTGTTGAGATCCAGAAGCACCATCGCCAAGTCAC | 1253 |
|  | CxrA-P-R | GGCCGACGACTTGAGAAACT |  |
|  | CxrA_R94A_-F1 | TCTCAAGTCGTCGGCCATGACCACGCCGA | 283 |
|  | CxrA_R94A_-R1 | GCCATCGTCACTGGGAGATGCATGGTCCGCGGTTGGGGT |  |
|  | CxrA_R94A_-F2 | TCTCCCAGTGACGATGGC | 2094 |
|  | CxrA_R94A_-R2 | CGTGGTGGTGGTGGTGGTGCCTGCCGTGAGCATGCAGCC |  |
|  | GFP-F | CACCACCACCACCACCACGTGAGCAAGGGCGAGGAG | 717 |
|  | GFP-R | TTACTTGTACAGCTCGTCCATG |  |
|  | CxrA-T-F | ACGAGCTGTACAAGTAAGGCAGCCCGCGGAGT | 905 |
|  | CxrA-T-R | TTGGTGATCTACATAAGCTCGTATCTGGTAGTGACGAAGCA |  |
|  | POX_d05452-RF | ATGTAGATCACCAAGTTGCACG | 2044 |
|  | POX_d05452-RR | AATCCACCGTCGCAATCGTCAA |  |
|  | POX_d05452-NF | ACGACTGGTAATCACGAATCA | 1557 |
|  | POX_d05452-NR | CGTCAAGGATATCGCCACTAA | 2028 |
| PCR confirmation | POX_d05452-F | ATGGTTGTCTTCAGCAAGGTTA | 1326 |
|  | POX_d05452-R | CTATGCCTGAGCAGCGAAAC |  |
|  | POX_d05452-LF | TGAGGGCAGGTGAAGTAG | 2458 |
|  | Ble-VR | ACTGAGGAATCCGCTCTTGG | 356 |
|  | Ble-VF | TGATAATAATGTCCTCGTTCCTGTC | 274 |
|  | POd05452-RR | AATCCACCGTCGCAATCGTCAA | 2044 |
| **Primers used for construction of *P. oxalicum* deletion mutants** | | | |
| ∆*prmt3*;*G418*^R+^ | PRMT3-LF | GAGTGGCAAGGAGCGTGG | 3069 |
|  | PRMT3-LR | CAATATCATCTTCTGTCGACTTCGAGTCTTTGATTTCAATGCC |  |
|  | PRMT3-RF | AGGTAATCCTTCTTTCTAGAAAGGCGACTCTCCTCCAGG | 3109 |
|  | PRMT3-RR | AGACCACCGATACCCATATCTT |  |
|  | PRMT3-NF | GGGACTGAAAGTGAGAATGAGAC | 1994 |
|  | PRMT3-NR | ACTTCCGAAAGGATTTGGCA | 2093 |
|  | G418-F | TCTAGAAAGAAGGATTACCTCTAAA | 1899 |
|  | G418-R | GTCGACAGAAGATGATATTGAAG |  |
| PCR confirmation | PRMT3-F | ATGACTCCCTCAACACAATCGA | 1903 |
|  | PRMT3-R | TCACTGAAGAGACCAGCGCTGGGCA |  |
|  | PRMT3-LVF | GCACCCAAGTTAAGCAAGCTT | 2968 |
|  | PRMT3-LVR | CAGTTCATTCAGGGCACCG |  |
|  | PRMT3-RVF | TCCAAGCAGCAAAGAGTGC | 3057 |
|  | PRMT3-RVR | TCCTGAGGTGATACCGCG |  |
| ∆*POX_a01843*;*G418*^R+^ | POX_a01843-LF | TTCATCCTTAACGGTCATAGCA | 3110 |
|  | POX_a01843-LR | GGTAATCCTTCTTTCTAGA TATTCAAGGTTGCTACCCTGC |  |
|  | G418-F | TCTAGAAAGAAGGATTACCTCTAAA | 1899 |
|  | G418-R | GTCGACAGAAGATGATATTGAAG |  |
|  | POX_a01843-RF | CAATATCATCTTCTGTCGACACAACCGACAAAGATGAGATTTC | 3112 |
|  | POX_a01843-RR | GATGTTTGGAAATGACCGACCC |  |
|  | POX_a01843-NF | CAGGAGATGAAAGAACGGATGA | 2132 |
|  | POX_a01843-NR | TATTTGCTGGTGGATGGTAGGT | 2177 |
| PCR confirmation | POX_a01843Gene-F | ATGGACTGGTCCGAGTCAAAG | 3684 |
|  | POX_a01843Gene-F | CTATTTCGCCCCAGCTTCAATA |  |
|  | POX_a01843-LVF | GATCGGCACGAATGGTAGA | 3732 |
|  | POX_a01843-LVR | GAGACCTACGAGACTGAGGAATC |  |
|  | POX_a01843-RVF | GAACTTAAGAAGGTATGACCGGG | 3575 |
|  | POX_a01843-RVR | ACCTTTCACGTGCCCCC |  |
| ∆*POX_g09341*;*G418*^R+^ | POX_g09341-LF | TCTTCAACGTGATCCGCCTG | 3130 |
|  | POX_g09341-LR | AGGTAATCCTTCTTTCTAGAGATTCTGGAAGATAAGAGACAAAA |  |
|  | G418-F | TCTAGAAAGAAGGATTACCTCTAAA | 1899 |
|  | G418-R | GTCGACAGAAGATGATATTGAAG |  |
|  | POX_g09341-RF | CAATATCATCTTCTGTCGACAGGATGCCGCTCCACATTCTGTA | 3168 |
|  | POX_g09341-RR | GAAAGCGGACCATTGCTGA |  |
|  | POX_g09341-NF | CAGTGACACCAGGCAGGCT | 2113 |
|  | POX_g09341-NR | GAGGCAGGGATGCTGTGGA | 1902 |
| PCR confirmation | POX_g09341Gene-F | ATGGATCTCATGGATGAAACCC | 4494 |
|  | POX_g09341Gene-F | TCATTCAAAATTTCCTACGCCA |  |
|  | POX_g09341-LVF | GCTCCTTGGTTGTGTAATTAGCA | 2955 |
|  | POX_g09341-LVR | GATCAACGGTCGTCAAGAGACCTA |  |
|  | POX_g09341-RVF | CGCCCAATAGCAGCCAGT | 3092 |
|  | POX_g09341-RVR | TTGGGGGAAAGCTTCGTG |  |
| **Primers used for construction of overexpression strain** | | | |
| O*POX_f07763*;*G418*^R+^ | POX_d05452-LF | TGAGGGCAGGTGAAGTAG | 2458 |
|  | POX_d05452-LR | TTTAGAGGTAATCCTTCTTTCTAGAGATGGACGAGACGGTACGAT |  |
|  | G418-F | TCTAGAAAGAAGGATTACCTCTAAA | 1899 |
|  | G418-R | GTCGACAGAAGATGATATTGAAG |  |
|  | POX_f07763-P-F | CAATATCATCTTCTGTCGACGGGCCTCTATGGCCTCAACCAATTACAG | 1281 |
|  | POX_f07763-P-R | TTTGACAACTCGAAATCCAATG |  |
|  | POX_f07763-F | GGATTTCGAGTTGTCAAAATGGGAGCCGGCGC | 876 |
|  | POX_f07763-R | GTGGTGGTGGTGGTGGTGGAAACCGACAAACTTGTTGAATC |  |
|  | GFP-F | CACCACCACCACCACCACGTGAGCAAGGGCGAGGAG | 717 |
|  | GFP-R | TTACTTGTACAGCTCGTCCATGC |  |
|  | POX_f07763-T-F | GACGAGCTGTACAAGTAATCGTCGAATCGGTCAAGACTG | 917 |
|  | POX_f07763-T-R | CAACTTGGTGATCTACATGTGGCGGCAATTTGACATCTAC |  |
|  | POX_d05452-RF | ATGTAGATCACCAAGTTGCACG | 2044 |
|  | POX_d05452-RR | AATCCACCGTCGCAATCGTCAA |  |
| PCR confirmation | POX_d05452-F | ATGGTTGTCTTCAGCAAGGTTA | 1326 |
|  | POX_d05452-R | CTATGCCTGAGCAGCGAAAC |  |
|  | POX_d05452-LF | TGAGGGCAGGTGAAGTAG | 2458 |
|  | G418-VR | GCCCTGGGTTCGCAAAGATA | 246 |
|  | G418-VF | AATAATGTCCTCGTTCCTGTCTGC | 230 |
|  | POX_d05452-RR | AATCCACCGTCGCAATCGTCAA | 2044 |
| O*prmt3*;*G418*^R+^ | POX_b03158L-F | TTTGGCTGAGCAAGCAGGGAA | 2991 |
|  | POX_b03158L-R | AGGTAATCCTTCTTTCTAGAAGTTGGATGGAATGGACGATGGA |  |
|  | Ble-F | TCTAGAAAGAAGGATTACCTCTAAA | 1755 |
|  | Ble-R | TTACCGCTGTTGAGATCCAG |  |
|  | PRMT3P-F | TTACCGCTGTTGAGATCCAGAACATGGAATCCCTCGTACTGC | 1441 |
|  | PRMT3P-R | GATTGTGTTGAGGGAGTCATTTCGAGTCTTTGATTTCAATGCC |  |
|  | PRMT3-F | ATGACTCCCTCAACACAATCGA | 1638 |
|  | PRMT3-R | AGCTCCTCGCCCTTGCTCACCTGAAGAGACCAGCGCTG |  |
|  | GFP-F | GTGAGCAAGGGCGAGGAG | 717 |
|  | GFP-R | TTACTTGTACAGCTCGTCCATGC |  |
|  | POX_c04083T-F | TGGACGAGCTGTACAAGTAATCGTAGGATAAACTTTGATCCAATC | 1067 |
|  | POX_c04083T-R | CTCTCCTCAAAATGGACCGCTTCATCTAGTACAATTGCACCGAGA |  |
|  | POX_b03158R-F | GCGGTCCATTTTGAGGAGAGCAC | 3258 |
|  | POX_b03158R-R | CATCTGCGTCCAACTCTTTCTGC |  |
| PCR confirmation | POX_b03158-F | AAGTCCTTCACTCTGCTCACA | 1425 |
|  | POX_b03158-R | TTACGAGGACTTGGCCTTGGC |  |
|  | POX_b03158LF | GTGGCTCCCAGAACAACAAC | 2132 |
|  | Ble-VR | ACTGAGGAATCCGCTCTTGG | 2488 |
|  | Ble-VF | TGATAATAATGTCCTCGTTCCTGTC | 8394 |
|  | POX_b03158RR | CATCTGCGTCCAACTCTTTCTGC | 3258 |
| O*cxrA-gfp*;*G418*^R+^ | POX_d05452-LF | TGAGGGCAGGTGAAGTAG | 2458 |
|  | POX_d05452-LR | TTTAGAGGTAATCCTTCTTTCTAGAGATGGACGAGACGGTACGAT |  |
|  | G418-F | TCTAGAAAGAAGGATTACCTCTAAA | 1899 |
|  | G418-R | GTCGACAGAAGATGATATTGAAG |  |
|  | CxrA-P-F | ATCTTCTGTCGACGGGCCAGCACCATCGCCAAGTCAC | 1253 |
|  | CxrA-P-R | GGCCGACGACTTGAGAAACT |  |
|  | CxrA-F | TCTCAAGTCGTCGGCCATGACCACGCCGATCTCTAC | 2380 |
|  | CxrA-R | GTGGTGGTGGTGGTGGTGCCTGCCGTGAGCATG |  |
|  | GFP-F | CACCACCACCACCACCACGTGAGCAAGGGCGAGGAG | 717 |
|  | GFP-R | TTACTTGTACAGCTCGTCCATG |  |
|  | CxrA-T-F | ACGAGCTGTACAAGTAAGGCAGCCCGCGGAGT | 905 |
|  | CxrA-T-R | GCAACTTGGTGATCTACATGCGTATCTGGTAGTGACGAAGCA |  |
|  | POX_d05452-RF | ATGTAGATCACCAAGTTGCACG | 2044 |
|  | POX_d05452-RR | AATCCACCGTCGCAATCGTCAA |  |
|  | POX_d05452-NF | ACGACTGGTAATCACGAATCA | 1557 |
|  | POX_d05452-NR | CGTCAAGGATATCGCCACTAA | 2028 |
| PCR confirmation | POX_d05452-F | ATGGTTGTCTTCAGCAAGGTTA | 1326 |
|  | POX_d05452-R | CTATGCCTGAGCAGCGAAAC |  |
|  | POX_d05452-LF | TGAGGGCAGGTGAAGTAG | 2458 |
|  | G418-VR | GCCCTGGGTTCGCAAAGATA | 246 |
|  | G418-VF | AATAATGTCCTCGTTCCTGTCTGC | 230 |
|  | POX_d05452-RR | AATCCACCGTCGCAATCGTCAA | 2044 |
| ∆*prmt3*::*cxrA-gfp*;*G418*^R+^;*ble*^R+^ | POX_d05452-LF | TGAGGGCAGGTGAAGTAG | 2458 |
|  | POX_d05452-LR | GGTAATCCTTCTTTCTAGAGATGGACGAGACGGTACGAT |  |
|  | Ble-F | CTGGATCTCAACAGCGGTA | 1755 |
|  | Ble-R | TCTAGAAAGAAGGATTACCTC |  |
|  | CxrA-P-F | TACCGCTGTTGAGATCCAGAAGCACCATCGCCAAGTCAC | 1253 |
|  | CxrA-P-R | GGCCGACGACTTGAGAAACT |  |
|  | CxrA-F | TCTCAAGTCGTCGGCCATGACCACGCCGATCTCTAC | 2380 |
|  | CxrA-R | GTGGTGGTGGTGGTGGTGCCTGCCGTGAGCATG |  |
|  | GFP-F | CACCACCACCACCACCACGTGAGCAAGGGCGAGGAG | 717 |
|  | GFP-R | TTACTTGTACAGCTCGTCCATG |  |
|  | CxrA-T-F | ACGAGCTGTACAAGTAAGGCAGCCCGCGGAGT | 905 |
|  | CxrA-T-R | TTGGTGATCTACATAAGCTCGTATCTGGTAGTGACGAAGCA |  |
|  | POX_d05452-RF | ATGTAGATCACCAAGTTGCACG | 2044 |
|  | POX_d05452-RR | AATCCACCGTCGCAATCGTCAA |  |
|  | POX_d05452-NF | ACGACTGGTAATCACGAATCA | 1557 |
|  | POX_d05452-NR | CGTCAAGGATATCGCCACTAA | 2028 |
| PCR confirmation | POX_d05452-F | ATGGTTGTCTTCAGCAAGGTTA | 1326 |
|  | POX_d05452-R | CTATGCCTGAGCAGCGAAAC |  |
|  | POX_d05452-LF | TGAGGGCAGGTGAAGTAG | 2458 |
|  | Ble-VR | ACTGAGGAATCCGCTCTTGG | 356 |
|  | Ble-VF | TGATAATAATGTCCTCGTTCCTGTC | 274 |
|  | POX_d05452-RR | AATCCACCGTCGCAATCGTCAA | 2044 |
| **Primers used for yeast two-hybrid strain construction** | | | |
|  | CxrA_61-733_-F | ATGACCACGCCGATCTCTAC | 2022 |
|  | CxrA_61-733_-R | TCACCTGCCGTGAGCATG |  |
|  | CxrA-AD-F | TACGACGTACCAGATTACGCTCATATGACCACGCCGATCTCTAC | 2202 |
|  | CxrA-AD-R | TTCACTGGCCTCCATGGCCATATGTCACCTGCCGTGAGCATG |  |
|  | cxrA_R94A_-R1 | GCCATCGTCACTGGGAGATGCATGGTCCGCGGTTGGGGT | 283 |
|  | cxrA_R94A_-F2 | TCTCCCAGTGACGATGGC | 2094 |
|  | CxrA-BD-F | CATGGAGGCCGAATTCCCCGAGAGAGAATTATTG | 2202 |
|  | CxrA-BD-R | GCAGGTCGACGGATCCTTATGGATGTGACGCGGATAG |  |
| **Primers used for RT-qPCR assay** | | | |
|  | PD-CxrA_61-733_-F | AGGGGCCCCTGGGATCCCCGATGACCACGCCGATCTCTAC | 2022 |
|  | PD-CxrA_61-733_-R | GGCCGCTCGAGTCGACCCGGTCACCTGCCGTGAGCATG |  |
|  | PD-PRMT3-F | CCATGGCTGATATCGGATCCATGACTCCCTCAACACAATCGA | 1641 |
|  | PD-PRMT3-R | CAAGCTTGTCGACGGAGCTCTCACTGAAGAGACCAGCGCT |  |
|  | PD-POX_g09341-F | CCATGGCTGATATCGGATCCGTCGTCAATGCAGGTTTGAT | 606 |
|  | PD-POX_g09341-R | CAAGCTTGTCGACGGAGCTCTCATTCAAAATTTCCTACGC |  |
|  | PD-POX_a01843-F | CCATGGCTGATATCGGATCCCATACGAATCGGAAAGGGT | 726 |
|  | PD-POX_a01843-R | CAAGCTTGTCGACGGAGCTCCTATTTCGCCCCAGCTTCA |  |
|  | PD-POX_f07763-F | CCATGGCTGATATCGGATCCATGGGAGCCGGCGC | 876 |
|  | PD-POX_f07763-R | CAAGCTTGTCGACGGAGCTCTCAGAAACCGACAAACTTGTTG |  |
|  | PD-POX09495-F | CCATGGCTGATATCGGATCCATGCCGTCCAGGCAAGAA | 1332 |
|  | PD-POX09495-R | CAAGCTTGTCGACGGAGCTCTCACTGCTCTGCAGCAAAGTT |  |
|  | PD-POX02592-F | CCATGGCTGATATCGGATCCATGGTGCTTCAATTGCACGT | 1287 |
|  | PD-POX02592-R | CAAGCTTGTCGACGGAGCTCTCAGATACCGAGAAAGTCGGT |  |
|  | PD-POX02594-F | CCATGGCTGATATCGGATCCATGCTTGTGAAGAACTCCCTC | 1032 |
|  | PD-POX02594-R | CAAGCTTGTCGACGGAGCTCTTAAGCGTAGTCGAGTTCGA |  |
|  | RT-actin-F | CTCCATCCAGGCCGTTCTG | 169 |
|  | RT-actin-R | CATGAGGTAGTCGGTCAAGTCAC |  |
|  | RT-cbh1-F | GTACTTGCGATCCTGATGGG | 194 |
|  | RT-cbh1-R | CCACGGTGAAGGGAGACTTG |  |
|  | RT-cbh2-F | TACTACGCTTCCGAGGTTCAGAG | 215 |
|  | RT-cbh2-R | GTGTCCAGCCAAACGAAGG |  |
|  | RT-eg1-F | AACCTGGAAGAACGGCACC | 131 |
|  | RT-eg1-R | CCTTGTCACAGTCATCGGAGC |  |
|  | RT-PRMT3-F | AGGATGCTGATGCCGATTATT | 140 |
|  | RT-PRMT3-R | CCAGGACGACCTTGTCTTTGA |  |
| **Primers used for *in vitro* EMSA** | | | |
|  | β-tubulin-F | ACCTCACTTGCTCCGCTCTG | 412 |
|  | β-tubulin-R | FAM-ACAAACTTCATAGATGGAGTGGACA |  |
|  | PRMT3-P-F | AGACTTTTGTGGCGTGTGAG | 1208 |
|  | PRMT3-P-R | FAM-TTCGAGTCTTTGATTTCAATGCC |  |
